# From Nanoparticles to Microparticles in Plasma Electrolytic Oxidation: Transport, Incorporation, and Coating Performance

**DOI:** 10.3390/nano16181191

**Published:** 2026-09-21

**Authors:** Aydin Bordbar-Khiabani, Sara Bahrampour

**Affiliations:** 1 VTT Technical Research Centre of Finland Ltd., Tekniikantie 21, FI-02150 Espoo, Finland; 2 Independent Researcher, FI-00750 Helsinki, Finland

**Keywords:** plasma electrolytic oxidation, nanoparticles, microparticles, particle size effect, composite coatings, incorporation mechanism, corrosion resistance, bioactivity, suspension stability, functional surfaces

## Abstract

Plasma electrolytic oxidation (PEO) can incorporate suspended particles from the electrolyte into growing ceramic-like oxide coatings, but particle size strongly affects their transport, capture, transformation, and final distribution. This review compares nanoparticle and microparticle additions, with particular emphasis on suspension stability, electrophoretic and convective transport, access to discharge channels, interaction with transient oxide melts, and distribution across the coating thickness. Well-dispersed nanoparticles generally remain suspended more readily, access finer pores, and undergo more extensive interfacial reaction or dissolution, whereas microparticles are more strongly affected by sedimentation and are more often retained as discrete or partially fused inclusions in outer coating regions. These differences influence coating morphology, porosity, roughness, phase evolution, hardness, wear and corrosion resistance, photocatalytic activity, and biofunctionality. Primary particle size alone, however, is insufficient to predict coating behavior because agglomeration, surface charge, particle morphology, electrolyte chemistry, waveform, and reactor hydrodynamics can alter the effective size-dependent response. Meaningful comparisons of particle-assisted PEO coatings should therefore account for the effective in-bath particle size and suspension state.

## 1. Introduction

Plasma electrolytic oxidation (PEO), also known as micro-arc oxidation (MAO) or anodic spark deposition (ASD), is a high-voltage electrochemical surface treatment used to form thick, hard, ceramic-like oxide coatings on light alloys and valve metals [1,2]. It is widely applied to aluminum [3], magnesium [4], and titanium [5], and it is increasingly considered for other metals and alloys such as tantalum [6], zirconium [7], zinc [8], copper [9], and silicon [10]. PEO coatings typically improve corrosion and wear resistance, and they can also provide thermal and electrical functions depending on composition and processing conditions [1,2].

The key feature that distinguishes PEO from conventional anodizing is the electrical regime and the resulting discharge behavior [11]. Conventional anodizing uses relatively low voltages and produces a thin barrier layer that gradually passivates the surface. In contrast, PEO operates at much higher voltages, often with pulsed or bipolar waveforms [12]. Under these conditions, the growing oxide exceeds its dielectric breakdown limit, leading to the formation of plasma micro-discharges across the surface [13]. Because the discharges generate extreme local temperatures and pressures, they enable rapid melting, ejection, and resolidification of oxide [14]. As a result, PEO coatings can develop complex microstructures and crystalline phases that are difficult to achieve by low-voltage anodizing [15].

However, the same discharge-driven growth that enables thick ceramic layers also produces structural defects. Gas evolution, thermal shocks, and rapid quenching generate pores, voids, and microcracks, especially in the outer region of the coating [16]. Consequently, many PEO coatings show a dense inner layer and a more porous outer layer [17]. The inner region can act as a barrier, yet open porosity and crack networks can become pathways for aggressive ions. Open defects can therefore compromise long-term corrosion resistance, particularly in chloride-containing or otherwise aggressive environments [18,19]. In addition, high roughness and surface defects can increase friction and accelerate wear under sliding contact. For these reasons, improving coating compactness and controlling defect formation remain central goals in PEO research [20].

One approach to overcoming these limitations is to add solid particles to the electrolyte and form composite PEO coatings [21]. When particles are suspended in the electrolyte, they can be delivered to active discharge sites and become incorporated into the growing oxide [22]. This approach can reduce effective porosity by narrowing or filling discharge channels, and it can also introduce beneficial second phases [23]. Moreover, particle additions can provide targeted functions, such as improved hardness, reduced wear, enhanced barrier performance, catalytic activity, or bioactivity [24]. Particle-assisted PEO has therefore become an important route for tailoring coating architecture and performance.

A wide range of additives has been investigated, including ceramic particles such as MoS_2_ [25], SiC [26], ZnO [27], TiN [28], Mn_3_O_4_ [29], and TiB_2_ [30]; carbon-based materials such as graphene derivatives [31,32] and carbon nanotubes [33]; and low-friction solids such as PTFE [34]. In many systems, these particles promote a more compact morphology and improve corrosion and wear resistance [35]. At the same time, the final outcome depends strongly on particle chemistry, electrical parameters, electrolyte composition, and substrate alloy [36,37]. Therefore, a mechanistic view is necessary to interpret why the same additive can produce different results across studies.

Among the controllable variables, particle size is especially important because it changes both transport and incorporation. In this review, nanoparticles are considered particles with characteristic dimensions below approximately 100 nm, whereas particles larger than approximately 1 μm are referred to as microparticles [38]. Particles between approximately 100 nm and 1 μm are treated here as belonging to a submicron or intermediate size regime. Importantly, these limits should not be interpreted as sharp mechanistic boundaries. Rather, particle behavior changes progressively across the size spectrum as the relative importance of Brownian diffusion, electrophoretic migration, convection, gravitational settling, pore accessibility, and particle–plasma interactions evolves with increasing particle size. This size dependence extends beyond simple geometric scaling because it alters the balance between electrophoretic motion, convection-driven delivery, settling behavior, and the ability to enter discharge channels and fine pores [39]. It also affects surface reactivity and melting or softening behavior in the plasma environment [40]. Consequently, nanoparticles and microparticles often follow different incorporation routes and lead to different coating architectures [41].

Nanoparticles can be transported efficiently to the coating surface, and they can more readily penetrate discharge channels and near-surface porosity [42]. As a result, they are often distributed more uniformly across the coating thickness, particularly when dispersion is stable [17]. In addition, their high surface area can increase chemical reactivity within the discharge zone. Therefore, nanoparticles may promote in situ compound formation, stronger interfacial bonding, or partial dissolution and reprecipitation within the oxide [42]. In contrast, microparticles tend to be incorporated more sporadically and less uniformly [42]. They are often captured near discharge mouths or trapped as partially fused inclusions within the outer layer. Because their size can locally disturb discharge behavior and resolidification, microparticles may also increase roughness or promote localized defect formation in some cases [42].

Another distinction concerns reactive versus inert incorporation [23]. Smaller particles may soften, melt, or react more readily, especially if they participate in plasma-assisted reactions with electrolyte species or substrate-derived oxides. This can shift phase evolution and create new functional phases [29]. Larger particles, particularly those with high melting points or low reactivity, often remain largely unchanged and become mechanically trapped [43]. Even then, they can still influence microstructure by physically blocking pores or modifying heat flow and discharge dynamics [33]. However, the magnitude and uniformity of these effects can differ substantially from nanoparticle additions [41].

Nanoparticles can agglomerate in alkaline electrolytes, causing their effective size to shift into the micrometer range [42]. Consequently, poor dispersion can negate the expected benefits of nanoparticles and can lead to inconsistent incorporation [36]. Accordingly, dispersion strategy, stirring intensity, pH, surfactant use, and particle surface modification can determine whether nominal nanoparticles remain nanoscale during PEO [44].

This review therefore examines how effective particle size controls transport, incorporation, depth distribution, and the resulting properties of particle-assisted PEO coatings. Particular attention is given to electrophoretic and convective transport, particle capture during discharge and resolidification, particle–melt interactions, pore accessibility, and the consequences for coating morphology, phase composition, corrosion, wear, and functional performance.

## 2. PEO Coating Growth

### 2.1. Basic PEO Setup and Process Stages

**Figure 1:**
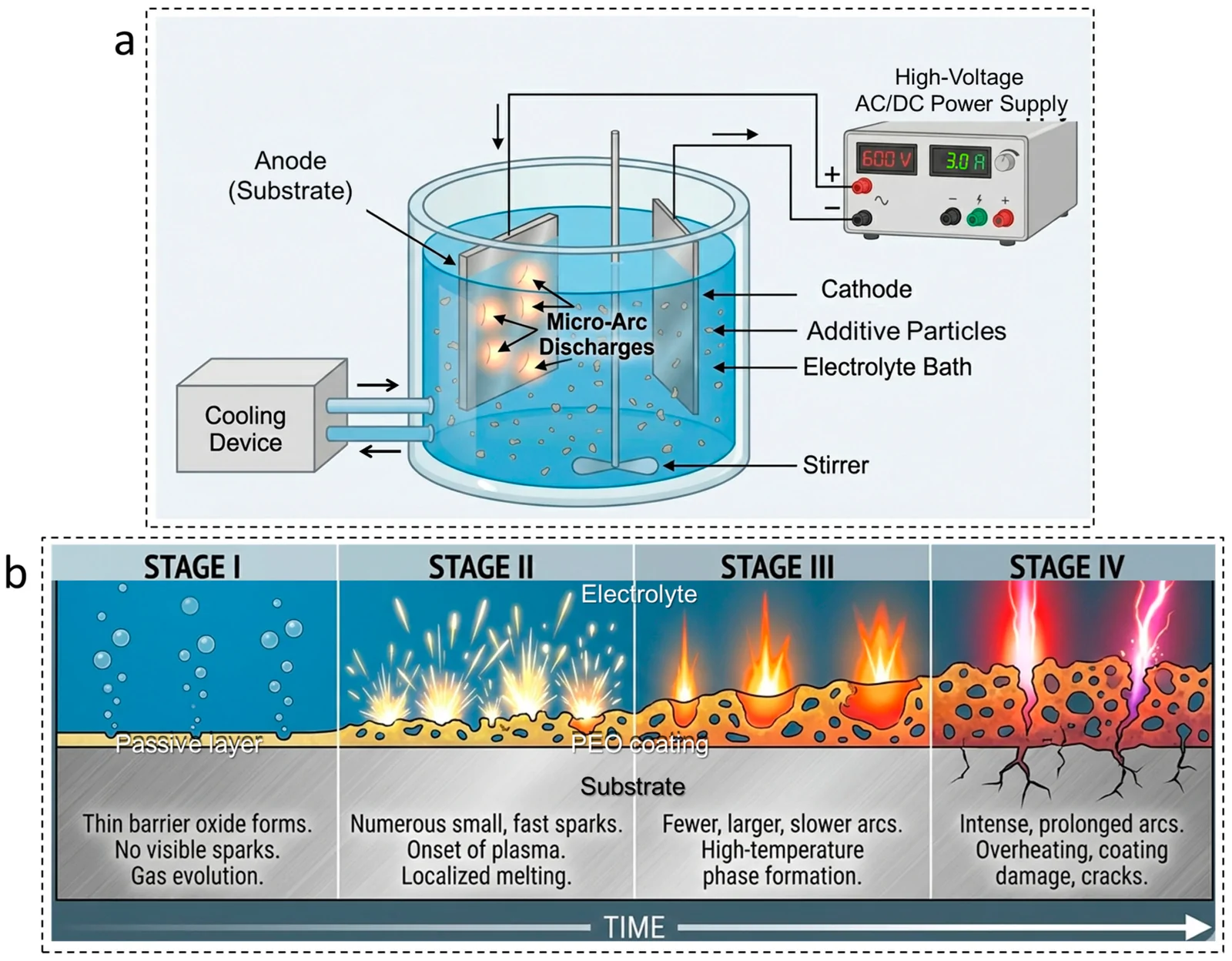
Schematic illustration of the PEO process and coating growth stages: (**a**) Experimental PEO setup showing the high-voltage power supply, substrate acting as the anode, cathode, electrolyte bath containing dispersed additive particles, stirring, and cooling system. (**b**) Typical evolution of the PEO coating with processing time: Stage I, formation of a thin barrier oxide with gas evolution and no visible sparks; Stage II, onset of plasma micro-discharges and localized melting; Stage III, development of larger and slower arcs accompanied by high-temperature reactions and rapid coating thickening; and Stage IV, intensified and prolonged discharges leading to increased porosity, microcracking, and potential coating damage.

PEO is an electrochemical surface treatment in which a high voltage is applied between the metal workpiece, which serves as the anode, and a cathode immersed in an electrolyte. Under these conditions, oxide coatings form on light metals such as Al, Mg, and Ti through a coupled mechanism involving conventional anodic oxidation, oxide dissolution, gas evolution, and plasma-assisted reactions [1,2]. Figure 1 schematically illustrates the PEO setup and the typical stages of coating growth. Unlike traditional anodizing, the applied voltage in PEO exceeds the dielectric breakdown strength of the growing oxide layer. As a result, the oxide undergoes localized dielectric breakdown, generating micro-arc discharges at the oxide–electrolyte interface.

Each micro-discharge forms within a transient gas or vapor envelope and creates a localized plasma channel in which temperatures of approximately 4900–10,000 K have been estimated spectroscopically, depending on the type and intensity of the discharge [45]. The intense electric field and Joule heating promote oxide melting, ion migration, and rapid thermochemical reactions. Within or near the discharge channel, substrate-derived metal cations react with oxygen-containing species originating from the electrolyte or the existing oxide. The principal electrochemical reactions associated with oxide and hydroxide formation during PEO are summarized in Table 1. These processes enable PEO to produce much thicker and harder ceramic-like coatings than conventional anodic films.

However, this plasma-assisted growth requires considerable electrical input. Typical PEO processing involves voltages of several hundred volts, while the applied current density may reach tens of A/dm^2^ depending on the substrate, electrolyte, electrical mode, and desired coating-growth rate [46]. Therefore, the process is characterized by a high local energy density that directly influences discharge behavior, coating growth, defect formation, and phase evolution.

The growth of PEO coatings generally proceeds through distinct stages of discharge evolution. In Stage I, a thin barrier oxide layer forms on the metal surface without visible sparks, and the process resembles conventional anodizing with simultaneous gas evolution. Once the dielectric breakdown voltage is reached, Stage II begins, and numerous small and fast-moving micro-discharges appear across the surface. These sparks indicate the onset of plasma-assisted reactions and localized oxide breakdown, while the rate of voltage increase often decreases because part of the supplied energy is consumed by discharge formation.

With further processing, the process enters Stage III, in which the discharges become fewer, larger, and more stable. The sparks move more slowly and tend to concentrate at specific sites, leading to increased local energy input and the formation of larger discharge craters [47]. This enhances oxide melting, resolidification, and high-temperature phase transformations. In alumina-based coatings, longer pulse-on times and higher discharge energies can promote the gradual transformation of metastable γ-Al_2_O_3_ into thermodynamically stable α-Al_2_O_3_ [48]. During this stage, the coating continues to thicken and its microstructure evolves.

Excessively prolonged treatment may lead to Stage IV, which is characterized by intense, long-lived arc discharges at isolated locations. These strong arcs can cause local overheating, enlarged discharge channels, coating damage, microcracking, and even substrate degradation [47]. Therefore, practical PEO processing is usually controlled to avoid or limit this regime. Overall, the progressive transition from uniform barrier-layer formation to micro-discharges and subsequently to larger micro-arcs governs coating thickness, porosity, phase composition, and functional properties.

### 2.2. Formation and Resolidification of Oxide Layers

Each micro-discharge in PEO can be regarded as a localized plasma micro-reactor that melts the surrounding oxide and, under sufficiently energetic conditions, a small portion of the underlying metal. The molten material can then be displaced or ejected toward the electrolyte and rapidly quenched. Repeated melting, outward transport, and resolidification contribute to the progressive inward and outward growth of the coating [49].

The expelled oxide resolidifies into characteristic pancake-like or crater-like features around the former discharge channel. A small central pore often remains, marking the discharge channel through which gases and molten material were transported. Consequently, the coating surface develops a porous, volcano-like morphology containing numerous micrometer-scale craters and central discharge pores. Repeated dielectric breakdown and material ejection are directly associated with the development of these cratered surface regions [48]. In addition, rapid cooling of the molten oxide promotes the formation of fine crystalline structures and metastable phases.

Alumina-based PEO coatings commonly contain γ-Al_2_O_3_ as a major metastable phase, together with variable fractions of α-Al_2_O_3_ and, in silicate-containing systems, phases such as mullite. Increasing pulse-on time, current density, or discharge energy generally promotes the γ-to-α transformation by increasing the thermal energy delivered to the coating [48]. However, phase evolution depends not only on treatment time but also strongly on waveform, current density, pulse duration, electrolyte composition, and local discharge characteristics.

Although individual discharges generate extremely high local temperatures, these events are short-lived and spatially confined. Therefore, when adequate electrolyte cooling and circulation are provided, the bulk substrate temperature generally remains limited despite transient temperature excursions at the oxide–metal interface. This localized heating–quenching cycle recurs across the surface, gradually increasing the coating thickness and defining its hierarchical microstructure.

PEO coating growth is governed by coupled electrochemical, plasma, and thermochemical processes. Electrochemical oxidation supplies oxygen-containing species at the anode, while plasma-assisted reactions within and around the discharge channels induce oxide melting, crystallization, and phase transformation. Isotopic-tracer investigations have demonstrated that oxygen species originating from water in the electrolyte can migrate toward the inner coating region, where fresh oxide forms through reaction with the aluminum substrate [50]. At the same time, intense current densities and Joule heating drive localized melting, whereas the surrounding electrolyte provides rapid cooling and solidification. This dynamic interaction at the plasma–oxide–electrolyte interface ultimately controls coating growth, porosity, elemental distribution, and microstructural evolution.

### 2.3. PEO Coating Architecture

The mature PEO coating typically exhibits a layered structure reflecting the sequence of oxide formation, dielectric breakdown, melting, transport, and rapid resolidification. A nanometric barrier layer lies directly at the metal–coating interface and originates from the anodic oxide formed before and during dielectric breakdown. In PEO-treated aluminum, studies report a barrier-layer thickness of approximately 200–500 nm [51]. A relatively compact intermediate layer and a more porous outer region typically overlie this barrier layer.

**Figure 2:**
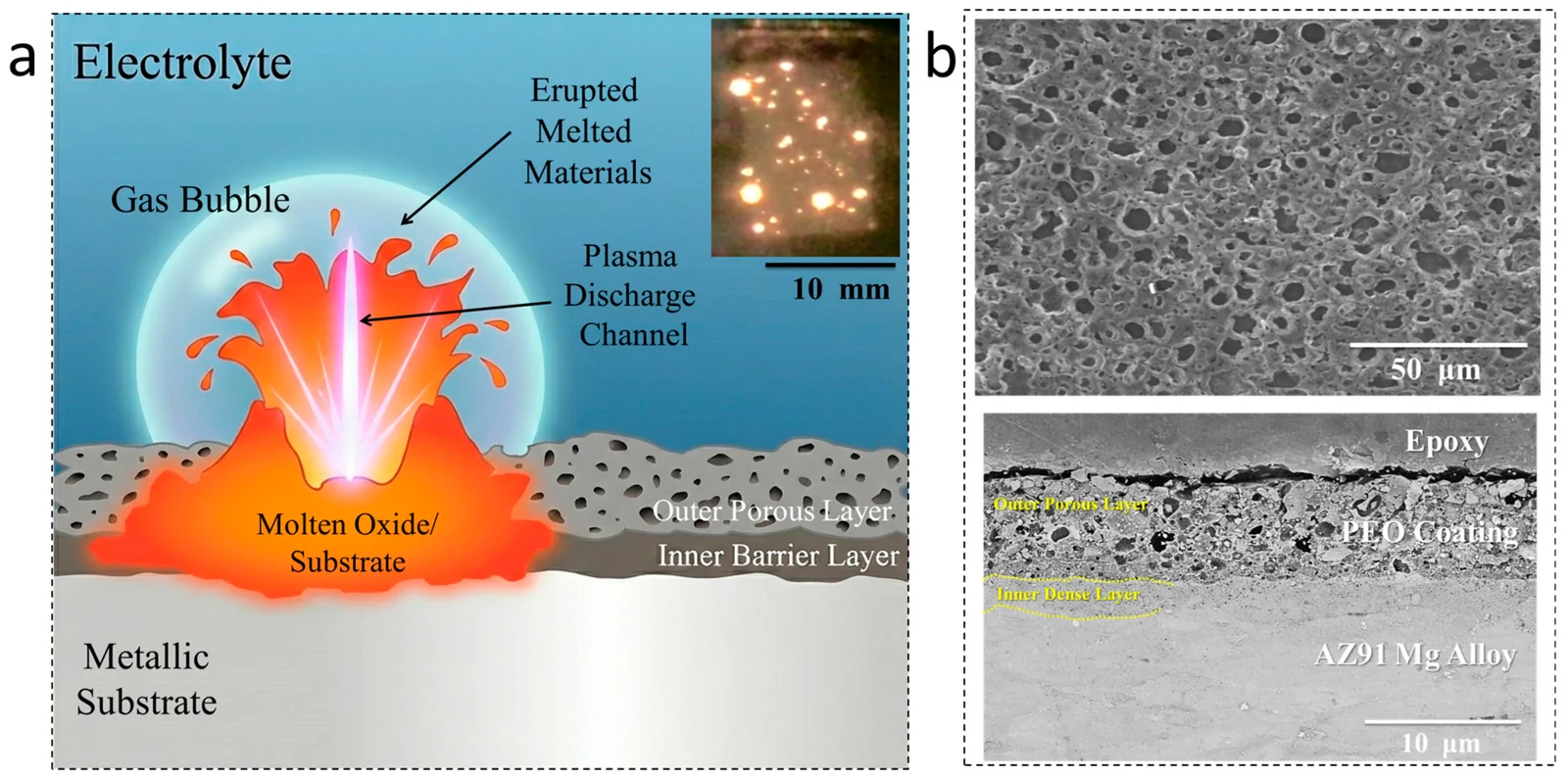
Plasma micro-discharge mechanism and typical microstructure of PEO coatings. (**a**) Schematic illustration of a plasma micro-discharge occurring within a gas bubble at the oxide–electrolyte interface, where the plasma channel locally melts the oxide and substrate and ejects molten material that rapidly solidifies to form the coating. The inset shows visible sparks during PEO processing. (**b**) SEM surface and cross-sectional images of a PEO coating on AZ91 Mg alloy showing the characteristic porous morphology and the layered structure consisting of a porous outer layer and a denser inner layer.

The barrier and compact inner regions are generally less porous than the outer coating and may contain amorphous oxide as well as fine crystalline phases. Because of their intimate contact with the substrate, these regions play important roles in coating adhesion, interfacial stability, load support, and corrosion protection. In contrast, the highly porous outermost region is often referred to as the loose or technological layer. This layer forms primarily from molten or partially molten oxide that is transported from discharge channels and rapidly resolidified on the coating surface, as schematically illustrated in Figure 2a.

The outer region is consequently characterized by higher roughness, interconnected porosity, discharge channels, and occasional microcracks. Quantitative investigations have shown that PEO coatings can contain substantial fine-scale, interconnected porosity, even when their cross-sections appear relatively compact under conventional microscopy [52]. Although this porous region can reduce barrier performance if left untreated, it may also provide beneficial features such as lubricant retention, mechanical interlocking with subsequent layers, increased surface area, or suitable sites for further functionalization.

Figure 2b shows the typical dense inner region and porous outer region of a PEO coating formed on AZ91 Mg alloy. Strong adhesion is generally associated with the conversion-growth mechanism of the coating, inward oxide growth, and chemical continuity at the metal–oxide interface rather than only with mechanical attachment [53]. Limited interdiffusion between substrate-derived and electrolyte-derived species may additionally contribute to interfacial stability.

In contrast, the porous outer layer generally contributes less to corrosion-barrier performance and is frequently removed, sealed, infiltrated, or otherwise modified during post-treatment. Therefore, reducing the relative thickness and interconnected porosity of the outer layer while preserving a sufficiently thick and compact inner region has become an important objective in PEO process optimization. Electrical regimes that promote soft sparking can improve the uniformity of discharge distribution and reduce the intensity of individual anodic discharge events [54]. These conditions can promote the formation of denser and less defective coatings.

### 2.4. Plasma–Electrolyte–Substrate Interactions

The interactions among plasma discharges, the electrolyte, and the substrate strongly influence the growth and properties of PEO coatings. The electrolyte not only completes the electrical circuit but also participates directly in coating formation. During discharge events, water and other electrolyte components near the plasma channel undergo dissociation and electrochemical reactions. These reactions contribute oxygen-containing species to oxide growth and generate gaseous products, particularly oxygen at the anodic surface [55].

The transient gas envelope surrounding an active discharge site creates conditions favorable for electrical breakdown and plasma formation. At the same time, plasma-assisted processes can ionize, melt, or transport substrate material and facilitate the incorporation of electrolyte-derived species into the growing oxide layer. As a result, many PEO coatings contain elements originating from the electrolyte. For example, silicate-based electrolytes commonly produce Si-enriched outer regions, whereas phosphorus introduced from phosphate electrolytes can be distributed more uniformly through the coating thickness [56].

Electrolytes may therefore be classified according to their contribution to coating formation. Some electrolytes primarily provide alkalinity and oxygen-containing species for substrate oxidation. Others contain anions, including silicates, phosphates, or aluminates, that participate in plasma-assisted reactions and form silicon-, phosphorus-, or aluminum-containing phases within the coating. A third group contains dissolved cationic additives, such as Ca^2+^ or rare-earth ions, which can act as dopants or reactants. Finally, particle-containing electrolytes include suspended solid additives that can become physically or reactively incorporated into the growing coating.

**Figure 3:**
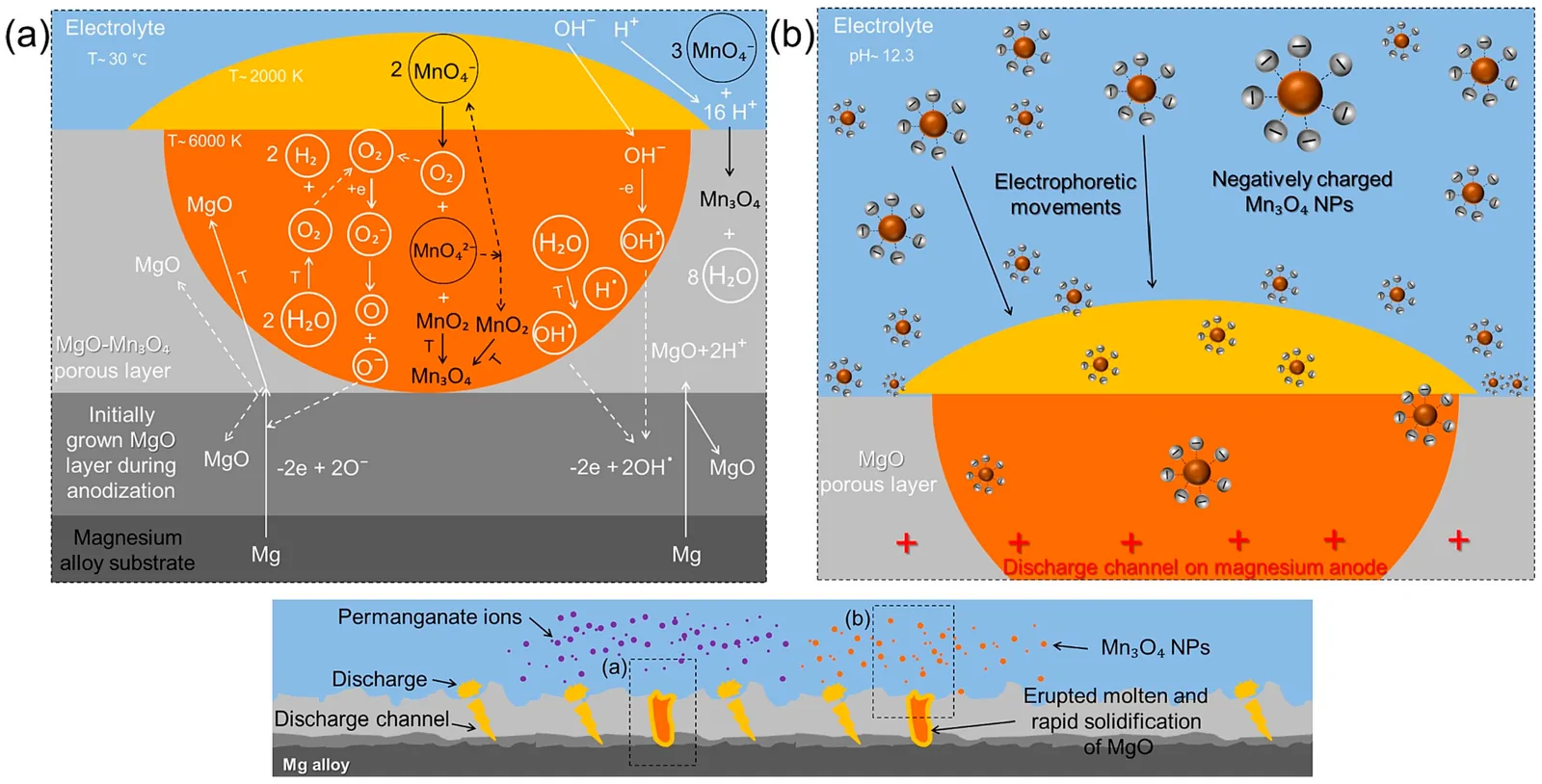
Schematic illustration of manganese species transport and incorporation during PEO of magnesium alloys. The lower image provides an overview of the coating-growth process, while the upper panels (**a**,**b**) are magnified views of the corresponding regions marked (**a**,**b**) in the lower schematic. (**a**) Magnified view of the plasma discharge region, showing chemical reactions occurring in the plasma discharge channel in a KMnO_4_-containing electrolyte, where permanganate ions participate in high-temperature plasma-assisted reactions; (**b**) Magnified view showing the electrophoretic transport of negatively charged Mn_3_O_4_ nanoparticles toward the anodic discharge sites and their subsequent incorporation into the molten oxide during coating growth. Reprinted with permission from Ref. [29] © 2024 Elsevier B.V.

During PEO treatment, electrolyte-derived anions and suspended particles carrying a negative surface charge can be transported toward active discharge sites by electrophoretic forces and convective flow. Once they reach the discharge zone, they may participate in plasma-assisted reactions or become incorporated into the coating through entrapment within the resolidifying oxide. For example, the addition of KMnO_4_ salt and Mn_3_O_4_ nanoparticles to the same base electrolyte can lead to the formation of MgO–Mn_3_O_4_ composite coatings on magnesium substrates. In this case, permanganate anions contribute manganese-containing species through discharge-assisted reactions, while Mn_3_O_4_ nanoparticles can migrate toward the anodic surface and become incorporated into the growing oxide layer. As illustrated in Figure 3, this process involves both reactive incorporation of electrolyte species and physical incorporation of nanoparticles during coating growth [29].

The composition and microstructure of the substrate also influence coating formation because PEO is fundamentally a conversion-coating process. Alloying elements and intermetallic particles can alter local electrical conductivity, oxidation kinetics, discharge initiation, and elemental incorporation into the coating. On AA2024 aluminum alloy, changes in electrolyte composition have been shown to produce substantial differences in coating-growth rate, surface morphology, phase formation, and elemental distribution [57]. Consequently, identical electrical conditions do not necessarily produce equivalent coatings on different alloys.

At the plasma–substrate interface, oxide formation competes with chemical and electrochemical dissolution and, under bipolar conditions, possible cathodic interfacial reactions. Rapid heating and cooling also generate transient thermal stresses within the growing oxide. The micro-discharge interface is therefore highly dynamic: oxide forms and melts within short time intervals, molten material is transported and rapidly solidifies, gas bubbles nucleate and collapse, and local chemical conditions fluctuate. If the discharges become excessively energetic, these processes can generate enlarged pores, voids, cracks, and other structural heterogeneities [47]. Controlling discharge intensity is therefore essential for producing uniform coatings with limited structural damage.

### 2.5. Influence of Process Parameters

**Figure 4:**
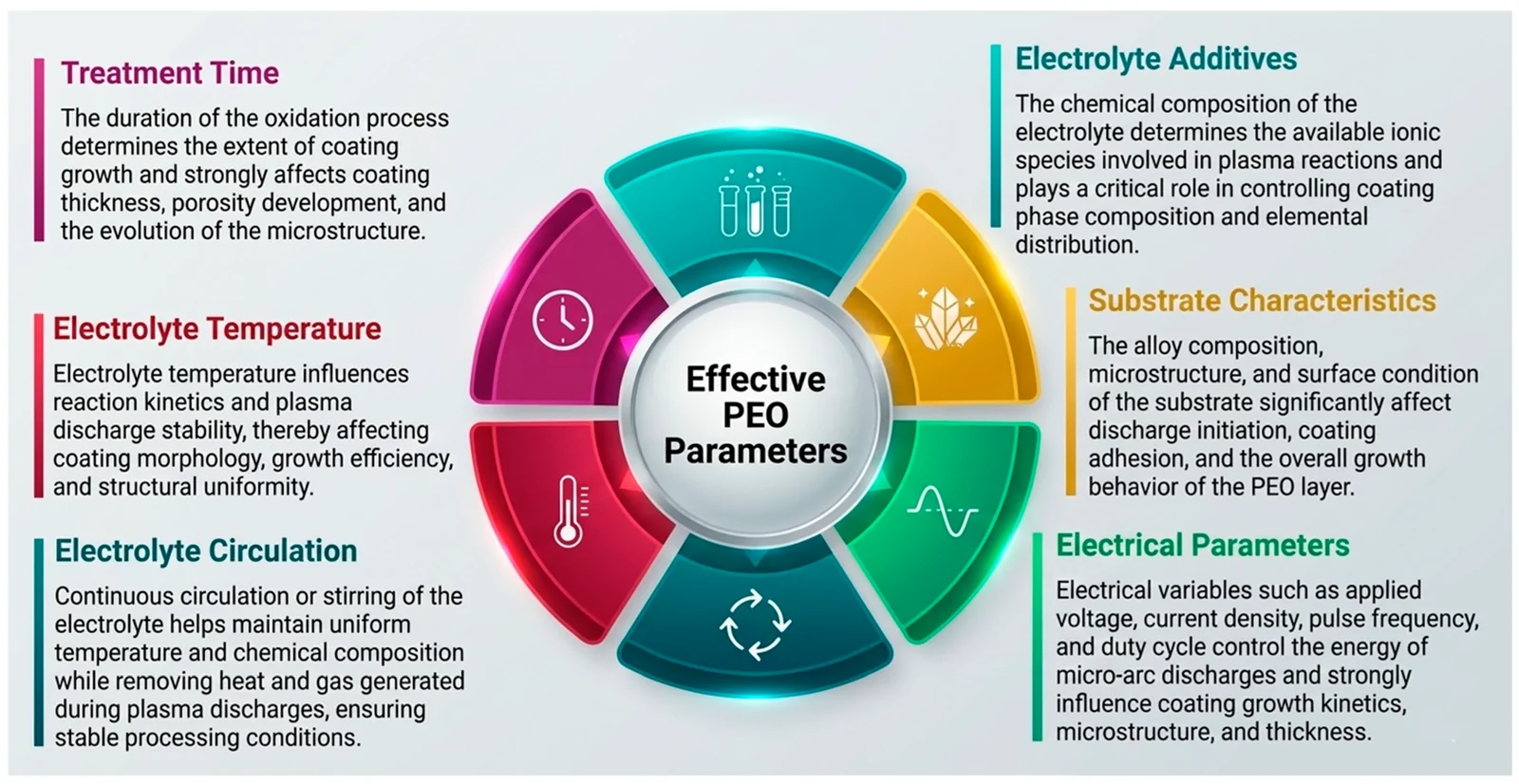
Schematic illustration of the key parameters affecting the PEO process and coating characteristics.

Figure 4 summarizes the principal processing variables governing PEO coating formation and properties. Applied voltage, current density, waveform, pulse frequency, and duty cycle directly control the energy, spatial distribution, and temporal behavior of micro-arc discharges and therefore affect coating-growth kinetics, thickness, porosity, elemental distribution, and microstructural development [46].

Under constant direct-current or constant-voltage conditions, discharges may become progressively more intense as the oxide layer thickens. This can result in large and long-lived arcs associated with excessive local heating. Such high-energy events can damage both the coating and the underlying substrate, leading to increased porosity, microcrack formation, and structural non-uniformity.

In contrast, pulsed and bipolar regimes periodically interrupt or reverse the applied polarization, allowing partial charge relaxation and thermal dissipation between successive anodic pulses. More generally, time-resolved studies of pulsed plasma discharges show that charge accumulation during a pulse modifies the local electric field and can generate a reverse electric field that governs discharge evolution and extinction [58]. Changing the current mode from unipolar to bipolar can suppress strong discharge events and promote the formation of a thicker and denser inner layer with fewer structural defects. Lower duty cycles can similarly generate a greater spatial density of lower-intensity micro-discharges and smaller discharge craters [46].

Appropriately selected bipolar conditions can lead to the soft-sparking regime, which is characterized by decreased anodic voltage, reduced light and acoustic emission, and a more uniform spatial distribution of discharges [54]. These changes are generally favorable for reducing excessive local energy input and producing denser and less defective coatings. Nevertheless, the effects of frequency, duty cycle, and waveform are interdependent and should not be interpreted independently of current density, electrolyte chemistry, substrate composition, and treatment duration.

Electrolyte composition is another major determinant of PEO behavior. The chemical composition of the electrolyte defines the ionic species available for electrochemical and plasma-assisted reactions and strongly influences breakdown behavior, coating-growth rate, phase formation, and elemental incorporation [57]. Variations in electrolyte chemistry, including the presence of silicates, phosphates, or aluminates, can substantially alter coating thickness, pore morphology, phase composition, and the spatial distribution of incorporated elements [56].

Electrolyte conductivity is also important because it affects the voltage distribution across the electrolyte and oxide layer and therefore influences the onset and intensity of discharge activity. However, the relationship is not universally linear because conductivity changes are commonly accompanied by changes in pH, ionic composition, dissolution kinetics, and species incorporation. Substrate characteristics must likewise be considered because alloy composition, intermetallic distribution, microstructure, and initial surface condition affect discharge initiation, coating adhesion, and overall coating-growth behavior. Consequently, even under nominally identical treatment conditions, different substrate materials may exhibit distinct oxidation responses and produce coatings with different structures and properties.

Other process variables, including electrolyte temperature, electrolyte circulation, and treatment time, further influence coating evolution and final performance. Because electrolyte temperature affects voltage–time behavior, reaction kinetics, discharge characteristics, growth efficiency, and morphology [59,60], it should be controlled and reported.

Electrolyte circulation or stirring helps maintain a more homogeneous temperature and chemical composition throughout the bath. It also assists in removing accumulated heat and gas bubbles and, in particle-assisted PEO, maintains the availability of suspended particles near the anodic surface. Together, these improvements in thermal and compositional homogeneity reduce local variations in discharge activity.

Treatment time is another critical variable because it determines the extent of coating growth and discharge evolution. Coating thickness commonly increases rapidly during the initial sparking stages and subsequently grows more gradually as the coating becomes thicker and more electrically resistive. However, excessively long treatment periods may increase the proportion of high-energy discharge events, enlarge surface craters, reduce coating-growth efficiency, and promote localized coating degradation [47,60].

## 3. Particle-Assisted PEO Process

In particle-assisted PEO, solid particles are dispersed directly in the electrolyte during coating growth. The electrolyte therefore serves both as an ionic medium and as a carrier for functional particles. These particles may include oxides, phosphates, silicates, carbides, nitrides, or bioactive compounds, depending on the target application. Suspending particles in the bath introduces solid coating constituents in addition to dissolved ionic species. As a result, the growing oxide layer can be enriched with phases that are difficult to form through substrate oxidation alone [61].

Particle incorporation involves transport through the electrolyte, delivery to the electrode surface, interaction with active discharge sites, and eventual capture or reaction within the growing oxide. Once near the surface, particles may adsorb on the outer layer, enter discharge cavities, or become captured during the refilling and resolidification of discharge pores [61]. Some particles remain chemically stable and are incorporated as discrete inclusions. Others undergo partial dissolution, reaction, or structural transformation under the local thermal conditions generated by micro-discharges [62]. Particle incorporation is therefore not merely mechanical embedding; it is coupled to dielectric breakdown, particle deposition and sintering, and oxide growth during PEO [63].

The presence of suspended particles can also modify the PEO process itself. Electrically active inclusions may alter coating resistance and redirect micro-discharges, while particle size can determine whether the inclusions accumulate on the coating surface or penetrate more deeply into the oxide. In some systems, particle incorporation promotes partial pore filling, coating densification, or the formation of additional functional phases. However, increasing the particle concentration does not necessarily improve the coating. Excessive particle loading can produce a more heterogeneous microstructure and increase the density of pores, cracks, or other coating defects [64]. A similar non-monotonic concentration dependence has been observed in other particle-modified coating systems, where an optimum additive content improves coating compactness and corrosion resistance, whereas excessive loading can promote agglomeration and reduce protective performance [62]. Accordingly, particle chemistry, concentration, size, surface charge, suspension conditions, electrolyte composition, substrate, and electrical regime must be considered together.

The incorporation pathway depends strongly on particle size. Under comparable dispersion conditions, smaller particles can more readily access fine discharge channels and pores, whereas larger particles are more strongly affected by sedimentation, inertia, and mechanical interception at the coating surface. Particle size also influences whether particles penetrate deeply into the coating, remain concentrated near the surface, or become embedded in the resolidifying oxide. A direct comparison of chromia particles with mean sizes of 69 and 351 nm showed that the finer particles penetrated more deeply and interacted closely with the alumina matrix and its pore network, whereas the larger particles accumulated mainly on the coating surface [64].

Thus, nano- and microparticle additions differ not only in scale but also in transport, access to discharge channels, interaction with the plasma, and contribution to coating structure. Among the studies surveyed here, the smallest reported primary particles were CeO_2_ nanoparticles below 5 nm, which were added to the electrolyte at a concentration of 5 g L^−1^ [65]. At the opposite end of the investigated range, hydroxyapatite particles with sizes up to 70 μm were dispersed in the PEO electrolyte at a concentration of 10 g L^−1^ [66]. Nevertheless, most of the particle-assisted PEO studies compiled for this review used additives smaller than 10 μm. These size-dependent aspects are discussed in the following sections.

## 4. Nanoparticles Versus Microparticles

### 4.1. Suspension Behavior: Zeta Potential, Agglomeration, and Sedimentation

Achieving a stable particle suspension in the PEO electrolyte is essential for reproducible particle transport and incorporation. Colloidal stability is commonly evaluated using the particle zeta potential, ζ, which reflects the electrostatic repulsion between suspended particles. As a practical guideline, |ζ| values of approximately 30 mV or higher are often associated with moderate electrostatic stability, while values above approximately 60 mV indicate strong resistance to agglomeration. By contrast, particles with ζ values close to zero generally exhibit a greater tendency to coagulate because the attractive van der Waals forces are insufficiently opposed by electrostatic repulsion. These thresholds are empirical guidelines rather than universal boundaries because suspension stability also depends on electrolyte ionic strength, particle concentration, surface chemistry, and steric interactions [67].

**Figure 5:**
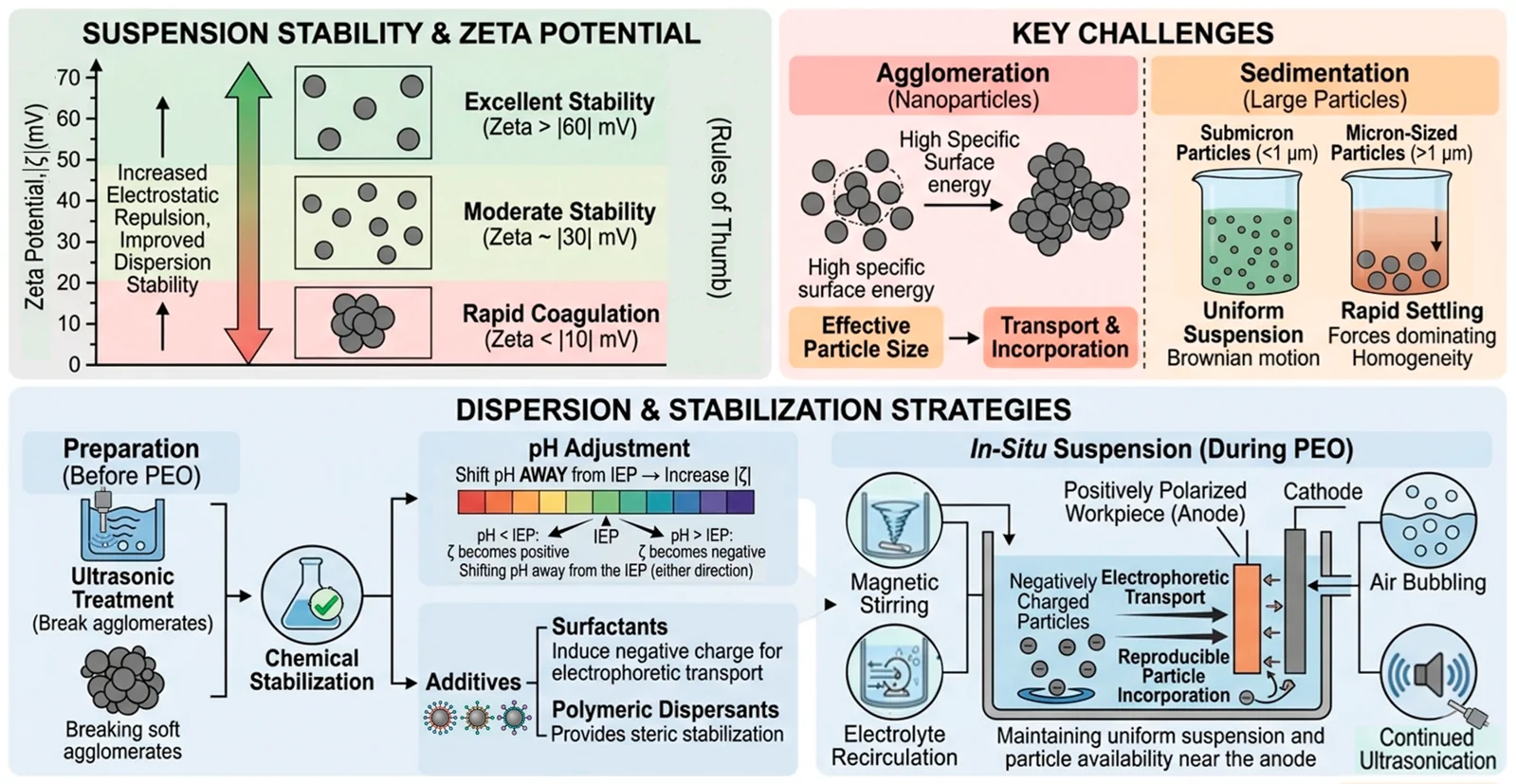
Schematic illustration of suspension behavior during particle-assisted PEO.

Electrolyte chemistry can therefore be adjusted to increase |ζ| and maintain particle dispersion during PEO. In alkaline silicate- or phosphate-based electrolytes, many oxide particles acquire a negative surface charge because the electrolyte pH lies above their isoelectric point. This behavior is favorable during the anodic period because negatively charged particles can migrate toward the positively polarized workpiece. Gnedenkov et al. reported that the addition of an anionic surfactant yielded a zeta potential of approximately −30 mV for the nano-additives, reduced their tendency to agglomerate, and facilitated electrophoretic transport toward the anode [68]. These interrelated aspects of suspension behavior are summarized schematically in Figure 5.

Agglomeration remains a major challenge for nanoparticles because their high specific surface area is associated with considerable surface energy. If electrostatic or steric stabilization is insufficient, nanoscale primary particles can rapidly form micrometer-scale agglomerates. The resulting clusters behave as larger particles and therefore exhibit altered diffusion, sedimentation, electrophoretic mobility, and incorporation behavior. Alumina nanoparticles dispersed in a PEO electrolyte have been shown to form suspension-dependent coating structures, demonstrating that nominal primary-particle size alone is insufficient to describe their behavior [69].

Several dispersion strategies can be used to maintain a homogeneous suspension. Ultrasonic agitation is commonly used during electrolyte preparation to disrupt weak agglomerates and may also be applied intermittently or continuously during PEO. Surfactants, low-molecular-weight dispersants, and polyelectrolytes can modify the particle surface charge or provide electrosteric stabilization. Alumina-sol additions have been used to introduce highly dispersed Al_2_O_3_-derived material into PEO coatings on AZ91D magnesium alloy [70].

Silica sols are particularly useful because their small colloidal particles can remain dispersed more readily than conventional silica powders. Silica-sol addition modifies the voltage response and oxide formation behavior of AZ91D magnesium alloy [71]. The same approach can produce hydrophobic anodic films when silica particles participate in surface and pore modification [72]. The incorporation of silica-sol particles also depends on the type of anions present in the electrolyte [73]. Increasing silicate concentration changes particle availability, electrolyte conductivity, and the structure of the resulting anodic layer [74]. Silica-sol-containing alkaline electrolytes have also been applied to PEO treatment of Mg-Li alloys [75].

Titania sols exhibit similar colloidal behavior, although their surface charge and stability depend strongly on pH and electrolyte composition. Titania-sol addition to borate electrolytes has been reported to improve the corrosion resistance of anodized AZ31 magnesium alloy [76]. More recent comparisons of soluble silicate, SiO_2_ particles, Si_3_N_4_ particles, and substrate-derived Si demonstrate that the chemical and physical form of the Si source strongly affects its availability for coating incorporation [77].

Electrolyte pH is particularly important because the difference between the operating pH and the particle isoelectric point strongly influences surface charge. Moving the pH away from the isoelectric point generally increases |ζ| and improves electrostatic stabilization. However, this relationship can be modified by specific ion adsorption, dissolved silicate or phosphate species, surfactants, and ionic strength.

Electrolyte aging can also compromise suspension reproducibility during PEO. The composition of a particle-containing electrolyte can change substantially during prolonged treatment or repeated processing cycles. With increasing bath age, the concentration and effective size distribution of suspended particles may change because of sedimentation, agglomeration, and progressive incorporation into the coating. At the same time, changes in pH, conductivity, ionic strength, and the accumulation or depletion of electrolyte-derived species can modify particle surface charge and therefore alter zeta potential and colloidal stability. Consequently, a suspension that is initially stable may exhibit different particle availability, transport, and incorporation behavior after extended use. To improve reproducibility, studies should report electrolyte age and reuse history, and parameters such as pH, conductivity, particle concentration, effective particle-size distribution, and zeta potential should ideally be monitored before and after successive processing cycles.

Continuous mixing is generally required when the electrolyte contains particles with high density, large effective diameter, or limited electrostatic stabilization. Clay-containing PEO electrolytes have shown that particle suspension, sintering, and coating formation are closely interconnected [78]. Current density also affects the interaction between suspended clay particles and active discharge sites [79]. In slurry electrolytes, mixing conditions directly influence coating uniformity because coarse particles can settle rapidly or accumulate within low-flow regions [80].

Particle sedimentation can be estimated using Stokes’ settling law when the particles are approximately spherical, the suspension is dilute, and the particle Reynolds number is sufficiently low [81].

(1) $$v_{s}=\frac{\left(\rho_{p}-\rho_{f}\right)gd^{2}}{18\eta }$$

where v_s_ is the terminal settling velocity, ρ_p_ is the particle density, ρ_f_ is the fluid density, g is gravitational acceleration, d is the particle diameter, and η is the dynamic viscosity.

Equation (1) shows that the settling velocity is proportional to d^2^. Thus, even a modest increase in effective particle diameter caused by agglomeration can produce a substantial increase in sedimentation rate. This quadratic size dependence is one of the main reasons why microparticles are more difficult to maintain in suspension than individual nanoparticles.

The Brownian diffusion coefficient of a spherical particle can be estimated using the Stokes–Einstein relationship [82]:

(2) $$D_{B}=\frac{k_{B}T}{3\pi \eta d}$$

where D_B_ is the Brownian diffusion coefficient, k_B_ is the Boltzmann constant, T is the absolute temperature, η is the dynamic viscosity, and d is the particle diameter.

The corresponding one-dimensional root-mean-square Brownian displacement over time t is [82]:

(3) $$L_{B}={\left(2D_{B}t\right)}^{1/2}$$

Equations (1) and (2) demonstrate that increasing particle diameter simultaneously increases the gravitational settling rate and decreases Brownian diffusivity. A 1.0 μm spherical particle with a density of approximately 2 g cm^−3^ in water at 25 °C has a calculated settling velocity of approximately 0.6 μm s^−1^ and a one-dimensional Brownian displacement of approximately 1.0 μm over 1 s. These two contributions are therefore of comparable magnitude under idealized conditions.

At the nanoscale, the balance shifts strongly toward Brownian motion. For a 50 nm spherical particle with the same assumed density of approximately 2 g cm^−3^ in water at 25 °C, Equation (1) gives a settling velocity of only approximately 0.0015 μm s^−1^ (1.5 nm s^−1^), whereas Equations (2) and (3) give a one-dimensional root-mean-square Brownian displacement of approximately 4.4 μm over 1 s. Thus, during a one-second interval, the characteristic Brownian displacement is nearly three orders of magnitude greater than the gravitational settling displacement. This comparison illustrates why individually dispersed nanoparticles can remain suspended much more readily than micrometer-sized particles. Brownian displacement, however, represents random motion rather than a net directional flux toward the workpiece.

For a 5 μm Al_2_O_3_ particle with a density of approximately 3.95 g cm^−3^, Equation (1) gives a settling velocity of approximately 0.045 mm s^−1^. Such a particle could settle approximately 8 cm within 30 min in a quiescent aqueous electrolyte. These estimates do not account for agglomeration, nonspherical geometry, concentrated-suspension effects, increased electrolyte viscosity, or hydrodynamic mixing.

Experimental studies support these size-dependent transport trends. Ag nanoparticles can be incorporated into anodic MgO layers when their suspension and delivery to the surface are adequately controlled [83]. CeO_2_ particles can modify coating morphology and electrochemical behavior, but their effect depends strongly on concentration and dispersion state [84]. Si_3_N_4_ particles require active mixing because their relatively high density and limited solubility favor sedimentation and non-uniform availability [85].

Consequently, magnetic stirring, electrolyte pumping, recirculation, gas bubbling, and ultrasonication are commonly used to preserve suspension homogeneity. The selected mixing method should be reported together with particle concentration, primary size, effective agglomerate size, zeta potential, electrolyte temperature, substrate orientation, and treatment time. Without these data, differences attributed to particle size may actually originate from differences in particle availability near the workpiece.

### 4.2. Transport Mechanisms

Once particles are dispersed in the electrolyte, their transport toward the anodic surface is governed by Brownian diffusion, convective flow, electrophoretic migration, and gravitational settling. The relative contribution of each mechanism depends on particle diameter, density, surface charge, electric-field strength, electrolyte viscosity, and the hydrodynamic conditions of the PEO cell.

For particles in the tens to hundreds of nanometers range, Brownian diffusion can contribute significantly to transport through the bulk electrolyte and the hydrodynamic boundary layer. Their random thermal motion promotes repeated contact with the electrode even when the bulk flow is limited. For micrometer-sized particles, the diffusion coefficient is much lower, while gravitational and inertial effects become increasingly important.

The relative importance of convection and diffusion can be expressed using the Péclet number (P_e_) [86]:

(4) $$P_{e}=\frac{UL}{D_{B}}$$

where U is a characteristic fluid velocity, L is a characteristic transport length, and D_B_ is the Brownian diffusion coefficient.

A low P_e_ indicates that diffusion contributes strongly to particle transport, whereas a high P_e_ indicates that convection dominates. Because D_B_ decreases with increasing particle diameter, P_e_ generally increases with particle size when the same velocity and length scales are used. However, no universal P_e_ can be assigned to a particular particle diameter because U and L depend on the reactor geometry, stirring rate, boundary-layer thickness, and gas evolution.

Electrophoretic migration must also be considered when the particles possess a measurable surface charge. For spherical particles, the Henry equation can be written [87]:

(5) $$\mu_{e}=\frac{\left(2\varepsilon_{r}\varepsilon_{0}\zeta f(\kappa a)\right)}{3\eta }$$

The electrophoretic velocity is then:

(6) $$v_{e}=\mu_{e}E$$

where μ_e_ is electrophoretic mobility, ε_r_ is the relative permittivity of the electrolyte, ε_0_ is the vacuum permittivity, ζ is the zeta potential, f(κa) is Henry’s function, κ is the inverse Debye length, a is the particle radius, η is dynamic viscosity, and E is electric-field strength.

Equations (5) and (6) show that increasing the magnitude of the negative zeta potential can increase migration toward the anodic workpiece, provided that the electric field extends through the particle-containing region of the electrolyte. However, the very high ionic strength of some PEO electrolytes compresses the electrical double layer and can modify both ζ and f(κa). Electrophoretic transport should therefore not be evaluated from particle charge alone.

A simple diffusion-length calculation illustrates the size effect. Based on Equations (2) and (3), the one-dimensional Brownian diffusion length of a 100 nm particle in water over 30 min is approximately 0.13 mm. For a 5 μm particle, the corresponding diffusion length is approximately 0.019 mm. Brownian diffusion can therefore assist nanoscale particles in traversing thin boundary layers, but it cannot normally transport them across macroscopic reactor dimensions without convection or electrophoretic migration.

The PEO process itself generates local fluid motion. Gas evolution, electrolyte heating, bubble growth and detachment, and thermal gradients near active discharge sites generate natural convection. Particle-melting experiments used to estimate plasma temperatures also illustrate the highly localized thermal conditions produced around discharge channels [88]. These local disturbances can temporarily entrain particles and direct them toward open pores and discharge cavities.

Nanoparticles generally follow changing fluid streamlines more closely because of their small relaxation time and low inertia. Carbon nanotubes have been transported into porous PEO alumina despite their anisotropic geometry, indicating that sufficiently small transverse dimensions can facilitate entrainment by electrolyte flow [89]. In contrast, larger and denser particles can slip relative to the surrounding liquid, settle through upward currents, or accumulate in low-flow regions.

Particle-containing electrolytes can also modify the process hydrodynamics and interfacial conditions. A multifunctional composite coating formed on magnesium alloy showed that suspended particles can influence pore filling, discharge behavior, and final coating structure simultaneously [90]. Electrolyte suspensions containing manganese- and nickel-bearing particles similarly produced oxide layers whose composition depended on particle transport and plasma-assisted conversion [91].

Natural convection generated during PEO is not always sufficient to maintain suspension homogeneity during long treatments. Active mixing is therefore commonly required. Degradation studies of clay-containing coatings showed that particle distribution and coating performance depend on whether the particles remain continuously available during coating growth [92].

The probability of incorporation depends not only on particle arrival at the workpiece but also on whether the particle encounters an open discharge channel, molten oxide, a growing conversion layer, or a suitable adsorption site. Crystalline TiO_2_-containing films on magnesium illustrate how suspended particles and particle-derived species interact with the coating during growth [93]. Titania-sol addition to aluminum electrolytes also changes the coating-growth response because the colloidal particles are continuously delivered to active regions [94]. TiO_2_-containing PEO coatings with photocatalytic activity similarly depend on transport of the titania source to discharge sites [95].

Al_2_O_3_ particles provide direct evidence of size-dependent capture. A mechanistic study on AM60B magnesium alloy showed that discharge-driven transport draws alumina particles into pores, where the resolidifying oxide immobilizes them [96]. Nano-Al_2_O_3_ addition to AZ91D produced more extensive modification of the coating than would be expected from simple outer-surface deposition [97]. Alumina-containing suspensions also demonstrate that agglomeration can reduce the uniformity and depth of particle incorporation [69].

ZrO_2_ nanoparticles have been studied using both alternating-current and direct-current PEO regimes. During AC PEO of magnesium, zirconia nanoparticles were found at the coating surface and within internal regions connected to discharge channels [98]. On aluminum, AC processing enabled zirconia nanoparticles to become incorporated into the growing oxide rather than remaining exclusively at the outer surface [99]. Under DC conditions, particle incorporation depended strongly on the access of the suspension to active discharge sites [100]. ZrO_2_-containing coatings on AZ91 magnesium further demonstrated that incorporated particles can alter the electrochemical response of the oxide layer [101].

Comparable transport dependence has been reported for other particle chemistries. Ag-containing TiO_2_ coatings require particle availability near active pores to develop antibacterial functionality [102]. SiC nanoparticles can improve coating compactness and tribological behavior when they remain suspended and reach the workpiece uniformly [103]. Particle-assisted composite PEO coatings on aluminum can additionally modify thermal-radiation behavior, indicating that functional-particle distribution is important throughout the treated area [104].

Particle morphology influences transport in addition to nominal particle size. Spherical particles can often be approximated using conventional settling, diffusion, and electrophoretic relationships, whereas elongated, tubular, platelet-like, or irregular particles may exhibit substantially different hydrodynamic drag, rotational motion, orientation, and interaction with fluid streamlines. High-aspect-ratio particles such as carbon nanotubes can align with local flow and access pores according to their transverse dimensions rather than their total length [89]. Similarly, halloysite nanotubes may have micrometer-scale lengths but nanoscale internal and external diameters, giving them transport and pore-access behavior that differs markedly from that of dense spherical microparticles [105]. Elongated particles may also become entangled or mechanically intercepted more readily than spherical particles. Consequently, particle size alone is insufficient to describe transport, and parameters such as aspect ratio, shape, flexibility, and effective hydrodynamic dimensions should also be considered when comparing particle-assisted PEO systems.

Cross-sectional electron microscopy of TiO_2_-Ag coatings has shown that particle-derived phases can be present at different depths in the oxide layer [106]. Such depth profiles may reflect transport of intact particles, transport of dissolved species, or repeated remelting and burial. Therefore, elemental mapping alone does not prove that the original particles penetrated intact.

Gravity can either assist or oppose delivery depending on substrate orientation. Sedimentation may increase coarse-particle availability on an upward-facing horizontal surface. For vertical components, downward-facing surfaces, or complex geometries, settling can create substantial spatial differences in particle concentration. Extrapolating results from small horizontal laboratory specimens to industrial components therefore requires explicit consideration of reactor hydrodynamics and component orientation.

### 4.3. Incorporation Mechanisms and Particle–Plasma Interactions

**Figure 6:**
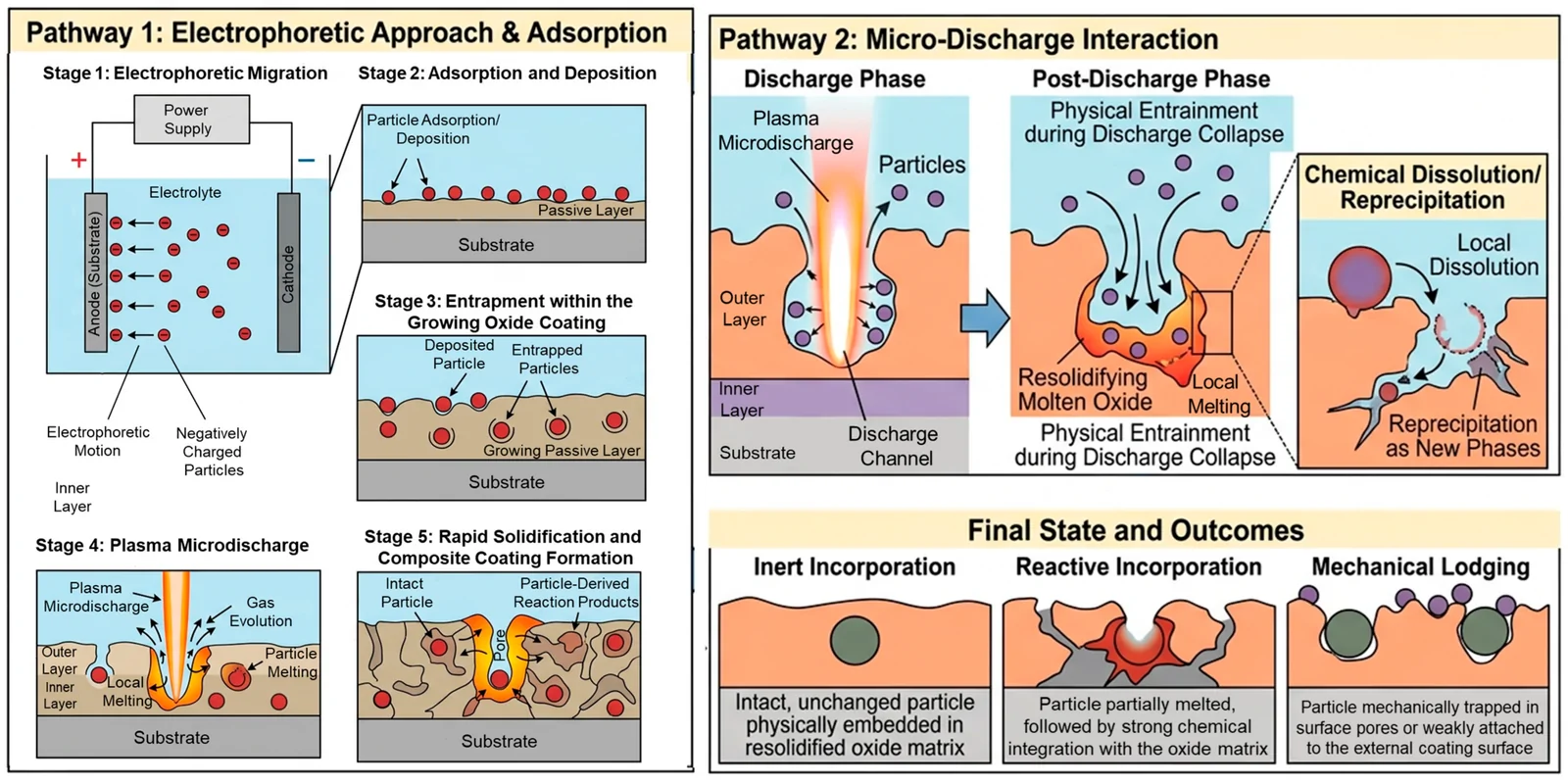
Schematic illustration of particle incorporation mechanisms during PEO.

Once particles reach the anodic surface, incorporation into the growing PEO coating can proceed through several interacting pathways, as summarized in Figure 6. These pathways include electrophoretic approach, adsorption, capture inside discharge cavities, entrapment within molten or resolidifying oxide, sintering, chemical reaction, dissolution and reprecipitation, and mechanical lodging within pores.

Electrophoretic transport toward the surface is favored when particles have a sufficiently negative zeta potential and remain individually dispersed. Equations (5) and (6) show that the particle velocity depends on ζ, electrolyte permittivity, viscosity, double-layer thickness, and electric-field strength [87]. Particles arriving at the surface may adsorb on the passive oxide and subsequently become buried by oxide growth or captured by a discharge event.

The incorporation of Al_2_O_3_ particles into PEO coatings on AM60B magnesium alloy has been described as a sequence involving particle adsorption, attraction toward active sites, entry into discharge channels, and entrapment during oxide resolidification [96]. Nanoparticle-containing coatings on titanium similarly demonstrate that field-assisted transport and plasma-assisted capture occur concurrently rather than as two independent processes [107].

During each discharge event, a transient plasma channel forms through the oxide. Localized heating, gas expansion, oxide melting, and material displacement create an open cavity. When the discharge collapses, electrolyte can flow back toward the hot region and carry suspended particles into the discharge pore. The particles may then be encapsulated by the rapidly resolidifying oxide.

Some particles remain largely unchanged and are incorporated as discrete inclusions. Other particles undergo reactive incorporation, resulting in changes in their initial composition or crystal structure. The pathway is controlled by particle size, thermal diffusivity, melting or decomposition temperature, solubility, electrical conductivity, wetting behavior, and chemical compatibility with substrate-derived oxides.

TiO_2_ particles provide several examples of reactive incorporation. Crystalline titania can be retained in magnesium-based PEO coatings while also participating in phase development [93]. Titania sol incorporated into aluminum PEO coatings can modify the oxide phase composition because its highly dispersed particles react more readily than coarse powders [94]. In phosphate electrolytes, titania-sol addition has also altered the composition and morphology of coatings produced on aluminum alloys [108]. Nano-TiO_2_ additions to 6063 aluminum have been associated with changes in coating growth, pore structure, and mechanical performance [109].

Zirconia can undergo both non-reactive and reactive incorporation. ZrO_2_ particles introduced during PEO of magnesium have been detected as retained particles together with transformed or reaction-derived phases [110]. AC processing can transport zirconia nanoparticles into connected internal regions of magnesium coatings [98]. Zirconia nanoparticles incorporated into aluminum under AC conditions can remain detectable within the oxide [99]. DC processing can produce different distributions because discharge duration and electrolyte access differ from those under AC conditions [100]. Zirconia-sol addition to AZ91D magnesium also modifies coating morphology and corrosion behavior [111]. Coarse ZrO_2_ particles incorporated into coatings on Al-Si alloy tend to remain more localized and are more likely to form discrete inclusions [112].

Calcium phosphate particles can be incorporated mechanically or can participate in dissolution and reprecipitation. Biphasic calcium phosphate-containing nanostructured films have been produced on magnesium by micro-arc oxidation [113]. Hydroxyapatite has also been introduced into porous TiO_2_ layers by combining micro-arc oxidation with electrophoretic deposition [114]. β-Tricalcium phosphate particles can become immobilized in PEO coatings on titanium [115]. One-step processes have produced hydroxyapatite-containing TiO_2_ coatings by combining plasma oxidation and particle deposition [116]. Porous titanium scaffolds can similarly be coated with microporous hydroxyapatite/TiO_2_ hybrid layers [117].

MgO/hydroxyapatite composite coatings on AZ31 demonstrate that calcium phosphate particles can be delivered into a pre-existing or simultaneously growing porous oxide [118]. On ZK60 magnesium, processing parameters affect both the amount of hydroxyapatite incorporated and the final coating morphology [119]. One-step formation of bio-composite coatings on magnesium further shows that particle dissolution, precipitation, and oxide growth may occur concurrently [120]. Direct comparisons of micro- and nanoscale hydroxyapatite additions have confirmed that particle size changes surface structure and biological response [121].

Silver-containing systems provide additional evidence of multiple incorporation routes. Ag nanoparticles can become embedded within anodic MgO layers and provide antibacterial activity [83]. In TiO_2_-Ag coatings, electron microscopy has shown complex distributions that reflect both intact-particle capture and Ag redistribution during growth [106]. Porous TiO_2_-Ag layers can therefore exhibit antibacterial activity even when the original particle morphology is only partially retained [102].

Mechanical lodging is more common for particles that are too large, chemically inert, or thermally stable to react extensively. Si_3_N_4_ particles can remain as discrete inclusions within the porous coating region [85]. Fe micrograins introduced during micro-arc oxidation primarily modify large pores and the external alumina region [122]. Graphite particles can be retained in surface pores and provide self-lubricating behavior [123]. Graphite-containing composite coatings produced using micro-arc plasma oxidation similarly rely on mechanical retention of the solid lubricant [124]. MoS_2_ particles incorporated into TiO_2_ coatings on Ti6Al4V can remain as a low-friction phase within the outer coating [125].

Particle heating during a discharge is extremely rapid but spatially non-uniform. Analysis of incorporated-particle melting has been used to estimate the local temperatures experienced during PEO [88]. Small particles can approach the surrounding melt temperature more rapidly than larger particles, while coarse particles may develop a reacted or softened shell around a relatively unchanged core.

#### 4.3.1. Effect of Particle Size on Plasma Discharge Characteristics

Suspended particles can influence breakdown voltage, voltage-time behavior, discharge density, apparent discharge size, discharge lifetime, optical emission, acoustic emission, and the transition to soft sparking. At an equivalent mass concentration, nanoparticles provide a much greater number of individual particles and a larger total interfacial area than microparticles. When well dispersed, they can alter electrical and thermal conditions over a large fraction of the coating surface. Microparticles instead create fewer and more spatially localized heterogeneities.

Whether particles intensify or suppress discharge activity depends on particle electrical conductivity, dielectric properties, concentration, agglomeration state, surface charge, and the electrical control mode. Multifunctional particle-containing coatings on magnesium demonstrate that the same additive can affect both discharge development and pore filling [90]. Suspensions containing manganese- and nickel-bearing particles likewise modify discharge chemistry as well as final coating composition [91].

Alumina-containing systems demonstrate the concentration dependence of these effects. Alumina-sol addition changes electrolyte behavior and the voltage response during coating growth [70]. Nano-Al_2_O_3_ particles can increase the number of potential interaction sites, but excessive addition can promote agglomeration and structural heterogeneity [97]. Superfine Al_2_O_3_ additions have also been reported to alter discharge distribution and coating compactness on AZ31 magnesium alloy [126]. Alumina nanoparticle suspensions can produce substantially different voltage-time curves depending on concentration and dispersion quality [69].

SiC nanoparticles can modify local electric fields and discharge behavior while becoming incorporated into the oxide. Their influence on AZ31 magnesium depends on whether the particles remain suspended and are delivered continuously to the growing layer [103]. Current density controls the interaction between SiC nanoparticles and molten alumina regions during micro-arc oxidation [127]. SiC-containing suspensions also show that changes in particle capture can modify both wear and corrosion behavior [128].

Titania nanoparticles affect discharge behavior through their dielectric properties, interfacial area, and chemical reactivity. TiO_2_ nano-additives in aluminate electrolytes modify coating formation on Mg-1Ca alloy [129]. Similar additions to magnesium PEO coatings alter discharge morphology and the resulting pore structure [130]. On AZ31 magnesium, TiO_2_ nanoparticle incorporation under bipolar current conditions produced concentration-dependent changes in corrosion, wear, and antibacterial behavior [131].

Particle additions may also modify thermal and functional responses. Composite PEO treatment on aluminum has shown that incorporated particles can alter both heat transfer and discharge-related coating development [104]. Al_2_O_3_/TiO_2_ nanocomposite coatings exhibit phase and morphology changes that depend on the interaction of both nanoparticle types with plasma events [132]. Particle-containing thermal-protection coatings on Al12Si piston alloy similarly develop through coupled discharge, melting, and particulate-capture mechanisms [133].

Conductive carbonaceous additives can produce particularly strong electrical effects. Carbon black nanoparticles can lower the breakdown voltage and promote an earlier transition to soft sparking when a suitable concentration is used [134]. Excessive carbon-black loading can instead promote agglomeration and deteriorate coating morphology. Multi-walled carbon nanotubes can also accelerate changes in the voltage response and soft-sparking transition [135]. Their elongated geometry and electrical conductivity produce behavior different from that of approximately spherical ceramic nanoparticles.

CNT-containing PEO coatings on aluminum have shown that nanotube addition alters both discharge behavior and coating corrosion resistance [89]. Carbon nanotubes incorporated during micro-arc oxidation of 6082 aluminum similarly modify coating morphology and electrical response [136]. Graphite particles can generate localized conductive regions and contribute to self-lubricating coating formation [123]. Graphite-containing electrolytes may also modify local discharge intensity as particle concentration increases [124].

The electrical control mode must be considered when interpreting these results. Under constant-current control, an increase in coating resistance generally requires a higher applied voltage. Under constant-voltage control, the same resistance increase can reduce current. Thus, apparently contradictory particle effects may arise from differences in power-supply mode rather than from particle chemistry alone.

Particle size may also influence the persistence of individual discharge sites. Fine particles captured inside a pore can be rapidly transformed, sintered, or buried. The affected channel may then become partially sealed, causing subsequent discharges to migrate elsewhere. A coarse particle lodged at a crater mouth may persist longer and concentrate the electric field around its perimeter. Depending on conductivity and wetting, it may promote repeated discharge activity, suppress the channel, or generate local cracking.

Pulsed processing can be used to improve nanoparticle capture. Ultra-high-frequency pulsing combined with Y_2_O_3_ nanoparticles increased the number of short-lived discharge events and promoted particle-enriched protective-layer formation [137]. TiO_2_ microparticle incorporation has also been shown to depend strongly on pulse frequency and whether direct-current or pulsed processing is used [138].

Recent mechanistic studies indicate that ceramic particles can interact with the oxide through several distinct scenarios, including inert entrapment, partial reaction, complete reaction, deformation, and phase transformation [96]. Therefore, particle diameter should not be interpreted independently of hardness, melting behavior, thermal conductivity, and the properties of the surrounding oxide. A recent comparison of soluble silicate, SiO_2_ particles, and Si_3_N_4_ particles further demonstrates that discharge response depends on both the physical size and chemical availability of the additive [77].

Future size-comparison studies should combine synchronized high-speed imaging, current-voltage transients, optical emission spectroscopy, acoustic measurements, and photodiode signals. Reporting only the final voltage or average current cannot reveal whether an additive changed discharge number, lifetime, energy, or spatial persistence. Useful descriptors include breakdown voltage, discharge count per unit area, lifetime distribution, apparent discharge diameter, optical intensity, soft-sparking transition time, and specific energy consumption.

#### 4.3.2. Effect of Particle Size on Interactions with Molten Oxide

The transient oxide melt surrounding a discharge determines whether an arriving particle is wetted, rejected, dissolved, sintered, reacted, or encapsulated. A first-order estimate of the size effect can be obtained from thermal diffusion. The characteristic time required for a particle to approach the local temperature can be expressed approximately as:

(7) $$t_{th}\approx \frac{d^{2}}{\alpha_{p}}$$

where t_th_ is the characteristic particle-heating time, d_p_ is the particle diameter, and α_p_ is the particle thermal diffusivity. Equation (7) is a scaling relationship rather than an exact solution because the actual heating process also depends on interfacial heat transfer, particle geometry, phase transformation, and the short lifetime of the discharge.

An order-of-magnitude estimate illustrates the strong size dependence of this thermal response. Taking αp ≈ 10^−6^–10^−5^ m^2^ s^−1^ as a representative range for ceramic particles, Equation (7) gives tth ≈ 0.001–0.01 μs (approximately 1–10 ns) for a 100 nm particle. For a 5 μm particle, the corresponding value increases to approximately 2.5–25 μs. Thus, increasing the particle diameter from 100 nm to 5 μm increases the characteristic internal thermal-diffusion time by a factor of approximately 2500. A nanoparticle with efficient interfacial heat transfer can therefore respond thermally within a microsecond-scale discharge, whereas a micrometer-sized particle may not equilibrate throughout its volume during a single short discharge.

The quadratic dependence on particle diameter means that nanoparticles heat and cool much more rapidly than compositionally identical microparticles. Their high surface-to-volume ratio additionally increases the relative rates of dissolution, interfacial reaction, and sintering. Experimental analysis of particle melting during PEO supports the importance of particle dimensions and thermal properties in determining whether a particle survives the plasma event [88].

For chemically compatible nanoparticles, rapid heating can produce partial or complete reaction with the oxide matrix. Al_2_O_3_ nanoparticles added during PEO of TC4 titanium alloy promoted Al_2_TiO_5_ formation and increased coating microhardness [139]. Nano-Al_2_O_3_ particles incorporated into magnesium coatings can similarly react with Mg-containing species or become sintered into the oxide [97]. Superfine Al_2_O_3_ additions on AZ31 have been associated with particle-assisted pore modification and composite-layer formation [126]. Al_2_O_3_/TiO_2_ nanoparticle mixtures can form composite phases whose final distribution differs from that of either starting powder [132].

ZrO_2_ particles illustrate the competition between phase retention and chemical transformation. Nanoparticles introduced into magnesium PEO coatings may retain ZrO_2_ while also participating in the formation of Mg-Zr-O reaction products [110]. AC PEO can produce both retained zirconia and transformed zirconia-containing regions [98]. On aluminum, the balance between intact incorporation and reaction differs under AC and DC conditions [99]. Under DC processing, prolonged localized heating can produce a distribution distinct from that generated by shorter alternating discharges [100]. Zirconia-sol additions can react more readily than coarse zirconia powders because the colloidal particles heat rapidly and provide a larger interfacial area [111]. Coarse ZrO_2_ particles in Al-Si coatings are more likely to remain partly unchanged and develop interfacial boundaries with the surrounding oxide [112].

TiO_2_ nanoparticles can dissolve, transform, or react with substrate-derived oxides. Titania nano-additives in Mg-1Ca coatings modify phase formation and corrosion behavior [129]. TiO_2_ nanoparticles introduced during MAO of magnesium can remain detectable while also changing the surrounding MgO-rich matrix [130]. Titania-sol particles in aluminum coatings can undergo more extensive reaction because of their small effective size [108]. Nano-TiO_2_ additions to 6063 aluminum alter both coating phase composition and mechanical performance [109]. Recent TiO_2_-containing coatings on AZ31 demonstrate that the beneficial concentration range is limited by the balance among incorporation, agglomeration, pore sealing, and defect formation [131].

Calcium phosphate particles have lower chemical stability in highly energetic discharge regions than many refractory ceramic particles. Biphasic calcium phosphate particles can partially decompose or react during formation of nanostructured films on magnesium [113]. Hydroxyapatite introduced into porous TiO_2_ can remain detectable while undergoing localized sintering or interfacial reaction [114]. β-Tricalcium phosphate can be retained in the coating while its interfaces react with titanium-derived oxide [115]. Hydroxyapatite-containing TiO_2_ coatings produced in a combined single-step process show both particle retention and matrix integration [116]. Microporous hydroxyapatite/TiO_2_ coatings on titanium scaffolds similarly contain regions with different degrees of particle consolidation [117].

MgO/hydroxyapatite composite layers demonstrate that larger hydroxyapatite particles may remain identifiable in the outer region while smaller or partially dissolved material contributes to deeper Ca- and P-containing regions [118]. Processing conditions on ZK60 magnesium affect whether hydroxyapatite particles are mechanically retained or incorporated through reaction-derived phases [119]. One-step bio-composite coating formation further indicates that particle dissolution and precipitation can occur during the PEO process [120]. These pathways explain why elemental enrichment cannot automatically be interpreted as intact-particle retention.

For a given composition, microparticles have greater thermal mass and can develop stronger internal temperature gradients during short discharge events. Their outer regions may soften, react, or sinter while their cores remain comparatively unchanged. Such nonuniform heating can produce partially fused inclusions. If wetting is favorable, the resolidifying oxide may form a mechanically interlocked shell around the particle. If wetting is poor, solidification shrinkage and thermal-expansion mismatch may create interfacial gaps.

Clay-containing systems provide evidence of particle sintering and partial reaction. Clay particles can soften or decompose in highly energetic regions while remaining more intact in cooler parts of the coating [78]. Increasing current density enhances thermal interaction but can also produce larger pores and coating damage [79]. During prolonged exposure, differences in particle bonding and coating connectivity influence degradation behavior [92].

Melt convection also affects nanoparticles and microparticles differently. Nanoparticles have low inertia and can follow rapidly moving molten material more closely. Microparticles may lag behind the melt, collide with channel walls, or be expelled with ejected oxide. Marangoni flow, capillary pressure, gas expansion, and vapor-recoil forces can redistribute particles over micrometer-scale distances during a single event.

Particle morphology also affects interaction with the transient oxide melt. Spherical or equiaxed particles generally interact more uniformly with the melt, whereas elongated or tubular particles can rotate, align, bridge pores, or become mechanically interlocked during resolidification. Carbon nanotubes and halloysite nanotubes are important examples because their transverse dimensions, rather than their total length, can control pore access and retention [105,136,140].

Platelet-like particles behave differently. Graphite platelets can align along pore walls and provide low-shear interfaces [124], whereas lamellar MoS_2_ can remain within the porous outer layer and act as a solid lubricant [125]. Particle dimensions should therefore be considered together with aspect ratio, flexibility, orientation, and particle–matrix interlocking.

Distinguishing reactive from non-reactive incorporation requires correlative evidence. Retention of the feed-particle crystal structure and morphology supports non-reactive incorporation. New interfacial phases indicate partial reaction. Disappearance of the original phase together with uniform elemental enrichment suggests extensive dissolution or transformation. Phase diagrams, thermal analysis, solubility measurements, cross-sectional TEM, diffraction, Raman spectroscopy, and chemical-state analysis should therefore be interpreted together.

#### 4.3.3. Effect of Particle Size on Penetration into Micro- and Nanopores

Particle penetration is partly controlled by geometric exclusion at the entrances to active discharge channels, pores, and cracks. Capture becomes progressively less probable as the effective particle or agglomerate diameter approaches the pore-throat diameter. However, there is no universal particle-size threshold because pore dimensions evolve with treatment time and vary with substrate, electrolyte, waveform, and discharge regime.

Nanoparticles can access narrower channels and more tortuous cracks than microparticles when they remain well dispersed. Direct penetration through an intact dense barrier layer should not be assumed. Suspended particles generally reach deeper regions only when discharge channels, interfacial defects, or repeated remelting events provide connected pathways.

Al_2_O_3_ incorporation studies show that particles can enter active pores and become immobilized during resolidification [96]. Zirconia nanoparticles have been observed within connected internal regions of coatings formed on magnesium [98]. Similar particles can reach deeper positions during AC PEO of aluminum [99]. Under DC conditions, their final depth depends on discharge persistence and access of fresh electrolyte to the channel [100]. ZrO_2_-containing coatings on AZ91 further demonstrate that internal particle-derived regions can affect barrier behavior [101].

Nanotube geometry may allow deeper retention even when particle length is relatively large. Carbon nanotubes have been detected throughout porous alumina layers because their nanoscale diameter permits access to channels that would exclude dense spherical microparticles [140]. Halloysite nanotubes can similarly enter large connected pores, although their micrometer-scale length may restrict penetration into highly tortuous pathways [105].

Microparticles are more likely to remain in large craters, open discharge channels, or the outer porous layer. TiO_2_ microparticles show strong dependence on pore dimensions and pulse conditions [138]. Coarse ZrO_2_ particles introduced during micro-arc oxidation of Al-Si alloy are predominantly localized near large pores and external coating regions [112]. Fe micrograins similarly modify surface porosity without necessarily penetrating the compact inner layer [122].

Si_3_N_4_ particles provide an example of size-dependent outer-layer retention. Their relatively large effective size and limited reaction with the oxide favor incorporation in accessible pores and surface regions [85]. SiC nanoparticles can penetrate smaller coating features when their suspension remains stable, although agglomeration can shift their behavior toward that of microparticles [128]. Agglomeration can eliminate the penetration advantage of nanoparticles. A powder consisting of 20 nm primary particles may behave as 2–10 μm clusters after exposure to an alkaline, high-ionic-strength electrolyte. Alumina nanoparticle suspensions demonstrate that the final effective size can differ substantially from the supplier-reported primary size [69].

Conversely, weak agglomerates may fragment under ultrasonic agitation, hydrodynamic shear, bubble collapse, or discharge-induced flow. Effective particle-size distributions should therefore be measured in the actual electrolyte before treatment, during suspension aging, and after processing. Zeta potential alone cannot establish whether the particles remain at their primary size.

Deep elemental enrichment does not necessarily prove penetration of intact particles. Dissolved particle-derived species may migrate inward and form new phases. The comparison of soluble silicate, SiO_2_ particles, and Si_3_N_4_ particles demonstrates that similar elemental depth profiles can arise from different transport and reaction pathways [77]. Phase-sensitive and nanoscale cross-sectional characterization is therefore required to distinguish intact particles from dissolved or transformed species.

**Figure 7:**
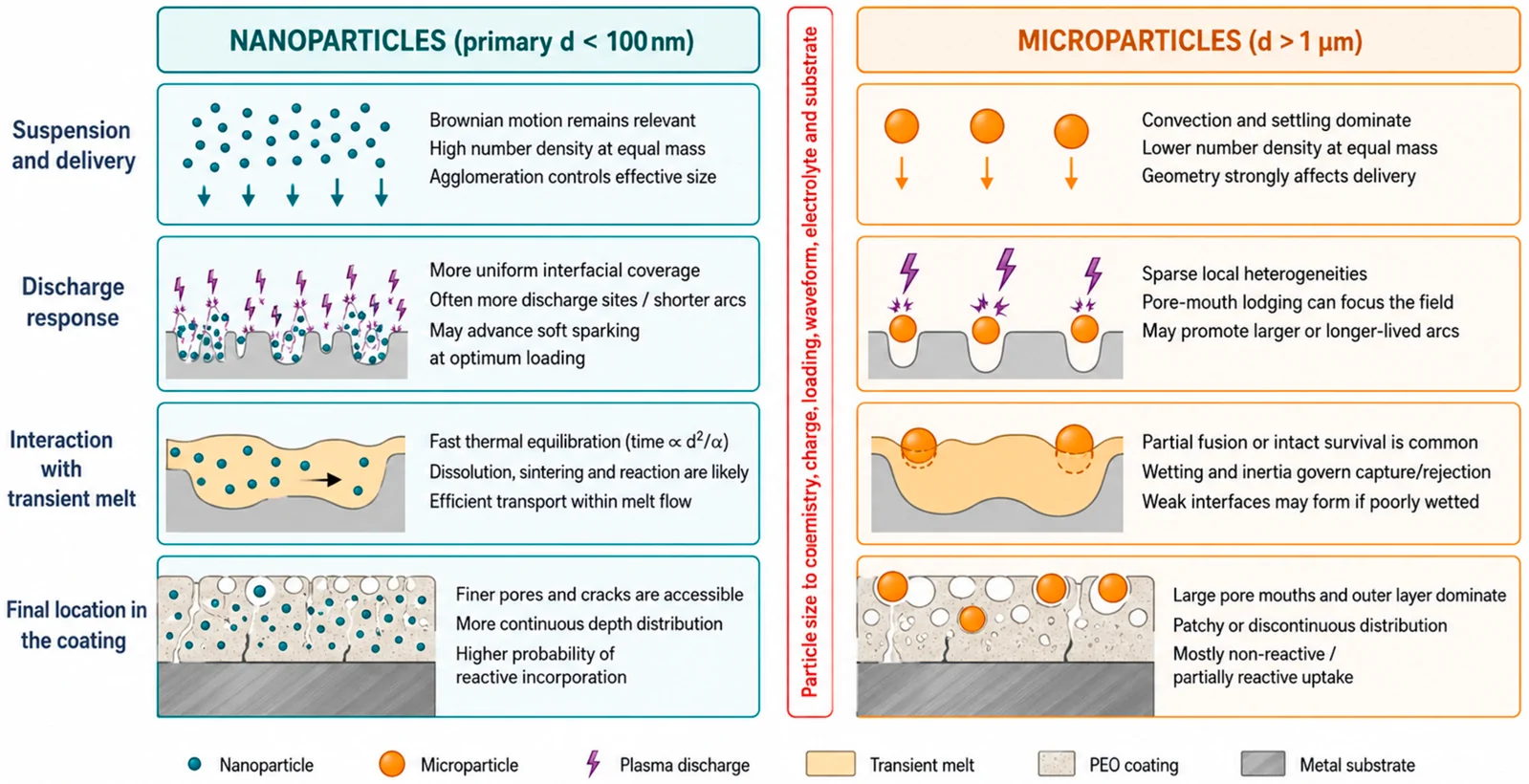
Size-dependent pathways linking particle suspension, discharge response, transient melt interaction, pore access, and final distribution in particle-assisted PEO coatings. The schematic emphasizes that primary size, effective agglomerate size, chemistry, concentration, waveform, electrolyte, and substrate are coupled variables.

Figure 7 summarizes the links among effective particle size, suspension behavior, transport, discharge interaction, pore access, and final particle location. The effect of particle size therefore emerges from coupled colloidal, hydrodynamic, electrophoretic, thermal, chemical, and geometric constraints. Nanoparticles are statistically more likely to remain suspended, reach active regions continuously, enter fine pores, and become distributed at greater depth. Microparticles are more likely to sediment and become localized in large pores or the outer layer. However, particle chemistry, agglomeration state, geometry, waveform, and coating architecture can produce important exceptions.

## 5. Size-Dependent PEO Coating Characteristics

### 5.1. Morphology, Porosity, and Roughness

Particle size modifies morphology through competing pore-filling, discharge-refining, and defect-generating effects. Well-dispersed nanoparticles can increase the number of active surface sites, promote smaller craters, enter fine channels, and become incorporated into the walls of the porous network. Within an optimal concentration range, these effects can reduce mean pore diameter, open porosity, and surface roughness [141]. The improvement is not guaranteed because agglomerates can behave as large inclusions, intensify localized discharges, and produce coarse nodules or cracks [142]. This concentration-dependent behavior is also observed in other particle-rich coating systems, where improved particle distribution and reduced porosity occur at an optimum modifier content, whereas excessive addition can promote agglomeration, pores, and cracks and thereby deteriorate corrosion resistance [143].

Microparticles are less uniformly distributed and tend to accumulate in large pores or on the outer surface. They can act as physical fillers when their dimensions are smaller than the pore mouth and wetting is adequate. Quasi-two-dimensional sericite microplates, for example, reduced coating defects and improved the compactness of MAO coatings on 2024 aluminum alloy [144]. Basalt particles smaller than 5 μm were also incorporated into thick PEO coatings, although their distribution and effect depended on particle composition and processing conditions [145]. Large or poorly wetted particles can instead increase roughness, leave interfacial voids, and detach during polishing or service.

Available direct comparisons generally indicate finer and more homogeneous coating structures when smaller particles are used. During PEO of AZ31B, alumina particle size and anode orientation jointly controlled particle uptake, corrosion resistance, and wear behavior, with nanoparticles showing greater sensitivity to substrate orientation than microparticles [146]. The effect should not be attributed to size alone because smaller particles also differ in number density, surface reactivity, agglomeration tendency, and suspension behavior.

Porosity should be characterized using more than a single two-dimensional area fraction. Plan-view SEM measures crater openings but does not resolve closed pores, undercut cavities, or connectivity to the substrate. Cross-sectional image analysis, focused-ion-beam serial sectioning, X-ray micro-/nanotomography, and electrochemical permeation provide complementary information. X-ray computed tomography has revealed internal pores and morphological features that cannot be reliably quantified from conventional surface or cross-sectional images [147]. Recent three-dimensional investigations have also identified interconnected subsurface pores between the inner and outer coating regions [148]. Reporting pore-size distributions, crack density, open and closed porosity, tortuosity, and roughness parameters such as Sa, Sq, Ssk, and Sku is therefore preferable to qualitative descriptions such as “more compact.”

### 5.2. Coating Thickness and Growth Rate

No universal relationship exists between particle size and PEO coating thickness. Particle addition can increase coating thickness by contributing external solid material, increasing the number of active sites, promoting inward or outward growth, or retaining material that would otherwise be ejected. Conversely, particles can decrease thickness by insulating the surface, blocking discharge channels, reducing individual discharge energy, or destabilizing the suspension. The outcome also depends on whether processing is controlled by current, voltage, or supplied energy.

Size influences these competing pathways through particle number, surface coverage, and capture probability. At equal mass concentration, nanoparticles provide a much larger population of particles and can modify a greater fraction of the surface. In Al_2_O_3_-sol-containing electrolytes, coating thickness increased up to an optimum sol concentration but decreased when further addition hindered discharge activity and coating growth [141]. Direct nano-/microparticle experiments similarly showed that particle uptake depends on size, gravity, and electrode orientation rather than nominal concentration alone [146].

Particle addition can also alter the voltage–time response and coating architecture without producing a monotonic change in thickness. Increasing anatase concentration during PEO of AA2024 modified process stability, phase composition, porosity, and particle-derived content, with excessive loading eventually destabilizing coating formation [142]. These results show why thickness should be interpreted together with mass gain, coating density, compact-layer fraction, and specific energy consumption.

For meaningful comparison, thickness should be measured at multiple positions and reported as a distribution, particularly for complex parts. Cross-sectional microscopy can distinguish compact and porous fractions, while dimensional-change or marker experiments can separate inward from outward growth. Gravimetry before and after removal of loosely attached deposits can quantify net mass gain. Coating volume and mass normalized by charge or energy provide more informative measures of growth efficiency than thickness alone.

### 5.3. Phase Composition and Crystallinity

Particle size affects phase evolution mainly through thermal equilibration, interfacial area, dissolution, and nucleation. Nanoparticles heat rapidly and provide a large reactive surface, favoring partial dissolution, reaction, or even complete consumption in the discharge zone. They may form spinels, titanates, silicates, phosphates, or mixed oxides with substrate- and electrolyte-derived species. They can also provide heterogeneous nucleation sites and refine crystallite size, although rapid quenching may preserve amorphous material or metastable phases.

Microparticles more often retain their original phases, allowing the starting phases to remain detectable by diffraction or Raman spectroscopy. Repeated discharge exposure can nevertheless sinter, oxidize, decompose, or transform their outer regions. Basalt particles introduced several particle-derived elements and phases into PEO alumina while also participating in high-temperature reactions [145]. Thus, classifying nanoparticles as reactive and microparticles as inert represents a general tendency rather than a universal rule.

The phase response also depends on particle-induced changes in discharge energy. A particle that reduces the energy of individual discharges may suppress high-temperature phases even when it is chemically reactive. Conversely, a field-concentrating inclusion may promote high-temperature transformation near the particle–matrix interface. In Al_2_O_3_-containing MAO coatings on Ti6Al4V, nanoscale α-Al_2_O_3_ promoted Al_2_TiO_5_ formation while improving coating thickness, microhardness, corrosion resistance, and wear behavior [149].

Particle-derived phases can also create functional heterostructures. Anatase additions produced multifunctional TiO_2_-containing coatings on AA2024 [142], while ZnO particles formed MgO/ZnO or MgAl_2_O_4_/ZnO structures with photocatalytic and optical activity [150,151]. Tungstate incorporation into Al_2_O_3_/ZnO coatings further modified phase composition and light absorption [152].

### 5.4. Depth Distribution and Elemental Mapping of Particles

Particle size directly affects depth distribution because finer particles can access smaller active pores, follow electrolyte or melt flow into connected internal regions, and undergo repeated burial, whereas coarse particles are more readily intercepted at large craters and remain concentrated near the outer surface. Direct comparison of nano- and micro-Al_2_O_3_ additions on AZ31B demonstrated that particle size and electrode orientation significantly affected incorporation and coating performance [146]. Increasing anatase loading on AA2024 also changed particle-derived elemental distribution through the coating thickness [142].

A uniform elemental map does not prove a uniform distribution of intact particles. Nanoparticles may dissolve in the plasma, react to form new phases, or release species that migrate independently. Conversely, discrete particles may lie below the spatial resolution or interaction volume of conventional SEM–EDS and appear as diffuse enrichment. Interpretation should therefore distinguish among intact-particle distribution, particle-derived elemental distribution, and reaction-product distribution. Silver nanoparticles, for example, were observed throughout PEO-grown TiO_2_ layers, but chemical and phase analyses were needed to establish their state and distribution [153].

Cross-sectional SEM–EDS provides a useful first assessment but is semi-quantitative and has a micrometer-scale interaction volume at commonly used accelerating voltages. Electron-probe microanalysis or wavelength-dispersive spectroscopy improves quantification; glow-discharge optical emission spectroscopy and time-of-flight secondary-ion mass spectrometry provide depth profiles; XPS sputter profiling identifies chemical states but may cause reduction or preferential sputtering; and TEM or STEM combined with EDS or EELS can resolve individual particles and particle–matrix interfaces.

Three-dimensional FIB–SEM or X-ray tomography can reveal particle clustering and pore connectivity when adequate compositional contrast is available. High-resolution X-ray tomography has demonstrated that apparently similar PEO regions may contain substantially different internal pore populations [147]. Micro-computed tomography has also resolved subsurface pore interconnectivity and its relationship with treatment time and duty cycle [148].

Quantitative studies should report particle-derived concentration as a function of normalized coating depth rather than only showing surface and cross-sectional maps. Useful descriptors include the outer-to-inner concentration ratio, depth containing 90% of the detected signal, cluster-size distribution, nearest-neighbour spacing, and fraction associated with open pores. Isotopically labelled or compositionally unique tracer particles can distinguish direct particle uptake from electrolyte-derived species. Correlative mapping before and after corrosion or wear can further determine whether deeply incorporated particles remain functional or are selectively released.

**Figure 8:**
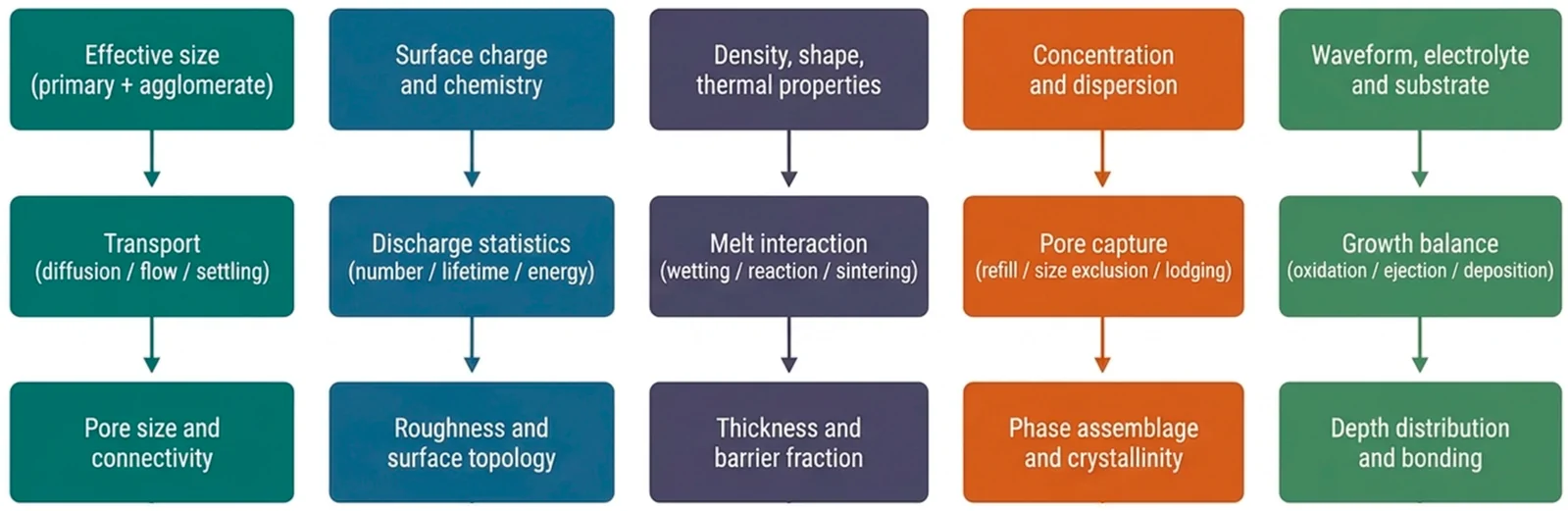
Causal map linking effective particle size and other feed/process descriptors to transport, discharge behavior, melt interaction, pore capture, and coating architecture.

Figure 8 links feed and process descriptors to particle transport, discharge behavior, melt interaction, pore capture, coating architecture, and performance, while highlighting chemistry, dispersion, electrical regime, substrate, and reactor geometry as major confounding variables.

### 5.5. Coating Performance

The performance of particle-assisted PEO coatings depends on particle size, chemistry, dispersion, concentration, and bonding with the oxide matrix. Well-dispersed nanoparticles can improve corrosion resistance by penetrating fine pores, narrowing discharge channels, increasing coating compactness, and participating in the formation of stable phases. Optimized Al_2_O_3_-sol addition increased coating thickness and hardness while improving the electrochemical response of AZ31B [141]. Incorporation of h-BN nanoparticles into PEO coatings on 2024 aluminum alloy similarly reduced defects and improved corrosion and wear resistance [154].

Microparticles can block large pores and introduce protective or corrosion-inhibiting phases, but their discontinuous distribution may leave connected crevices beneath lodged particles. Sericite microplates improved corrosion and wear resistance by filling defects and increasing coating compactness [144]. Excessive nanoparticle loading can also be detrimental because agglomeration, cracking, interfacial voids, or unstable discharge behavior can accelerate electrolyte penetration.

Mechanical and tribological performance can be enhanced through pore filling, phase hardening, crack deflection, load transfer, and solid lubrication. Nanoscale α-Al_2_O_3_ promoted Al_2_TiO_5_ formation and improved the hardness and wear resistance of coatings on Ti6Al4V, although excessive addition increased surface defects [149]. Nano-hBN additions reduced friction and wear by combining pore filling with solid lubrication [154,155].

Microparticles may also provide effective local reinforcement when they are sufficiently bonded to the oxide. Basalt-containing PEO coatings exhibited high wear resistance and a high friction coefficient suitable for applications requiring enhanced surface grip [145]. By contrast, poorly bonded particles may protrude, detach, initiate cracks, or act as abrasive third-body debris. Nano-additives generally provide more spatially uniform reinforcement, whereas the performance of microparticles depends strongly on particle retention and interfacial integrity.

Particle size also affects dielectric, thermal, and electrical functions. Ceramic nanoparticles can fill fine defects and increase dielectric continuity, whereas conductive or semiconducting particles may reduce coating resistance when a connected network develops. Microparticles are less likely to form continuous networks at the same mass loading but may concentrate the electric field at particle–matrix interfaces. Thermal behavior depends on intrinsic particle conductivity, porosity, interfacial bonding, and thermal-contact resistance. Fine particles can improve heat spreading when well connected, whereas numerous poorly bonded interfaces may increase phonon scattering. Hollow or low-conductivity particles can instead improve thermal-barrier behavior. More broadly, studies of particle-containing ceramic coatings show that high-temperature performance is governed not only by the intrinsic thermal properties of the constituent phases but also by particle distribution, interfacial bonding, pore sealing, and the formation of thermally stable reaction products [156].

Nanoparticles of TiO_2_, ZnO, WO_3_, and related compounds are effective for photocatalytic and optical applications because of their high specific surface area and extensive contact with the oxide matrix. TiO_2_-containing PEO coatings on magnesium showed particle-concentration-dependent photocatalytic performance [157]. ZnO particles incorporated into MgO- or MgAl_2_O_4_-based coatings increased photocatalytic activity [150,151]. Tungsten-doped Al_2_O_3_/ZnO coatings additionally showed modified light absorption and photocatalytic degradation behavior [152].

Particle-containing PEO coatings can also immobilize complex photocatalytic materials. Pure and Ce-exchanged zeolites were incorporated into PEO coatings on aluminum; zeolite composition and processing time affected photocatalytic and corrosion-protection performance [158]. Microparticles remain useful for optical scattering, pigmentation, luminescence, and thermochromic functions because they may retain a functional crystalline core during processing.

For biomedical coatings, hydroxyapatite, calcium phosphates, and Ag-containing particles can enhance mineralization, antibacterial activity, or biological interactions. Hydroxyapatite-containing porous TiO_2_ coatings promoted apatite formation and were associated with improved cellular responses [159]. Ag nanoparticles incorporated into TiO_2_ coatings provided strong antibacterial activity, although silver loading and release required control [153]. Smaller particles generally provide more homogeneous functional distributions, whereas microparticles may serve as slower-release reservoirs but can detach if weakly bonded. More broadly, the biological performance of implant surfaces depends strongly on their interfacial properties, since surface wettability, topography, and roughness influence protein adsorption and the subsequent foreign-body response [160]. Overall, the principal size-dependent tendencies, required conditions, and potential limitations associated with nanoparticle and microparticle additions in PEO are summarized in Table 2.

## 6. Challenges, Knowledge Gaps and Future Perspectives

### 6.1. Standardized Particle and Suspension Descriptors

Particle characterization remains uneven across the PEO literature. Supplier-reported primary size is often the only size descriptor, even though agglomeration in alkaline electrolytes can substantially shift the in-bath size distribution. Studies designed to isolate size effects should report primary and in-bath size distributions, zeta potential, particle shape and density, electrolyte pH and conductivity, mixing conditions, and suspension age. Measuring particle concentration before and after PEO would also permit incorporation efficiency to be estimated quantitatively.

### 6.2. Resolving Incorporation Mechanisms with Operando Diagnostics

Most proposed particle-incorporation mechanisms remain indirect because they are reconstructed from post-treatment microstructures. Direct discrimination among electrophoretic delivery, sedimentation, and pore refilling will require time-resolved measurements during PEO. Synchronizing high-speed imaging with electrical transients, and where feasible, optical or acoustic emission, would allow individual discharge events to be related to particle arrival and capture. Experiments that independently vary particle charge or specimen orientation would be particularly useful for separating electrophoretic and gravitational contributions.

### 6.3. Quantitative Three-Dimensional and Chemical-State Mapping

Conventional SEM–EDS identifies particle-derived elements but cannot reliably distinguish intact particles from dissolved or transformed species. Combining three-dimensional mapping with chemical-state or crystallographic characterization would provide a more reliable description of particle location and reaction state. Quantitative segmentation should also include uncertainty estimates and validation against manual or complementary measurements.

### 6.4. Durability, Particle Release, and Application-Relevant Testing

Most particle-assisted PEO studies assess freshly prepared coatings over relatively short test periods. Longer-term experiments are needed because particle detachment, dissolution of reaction products, and cyclic mechanical damage can alter both coating performance and particle release. Test conditions should therefore reproduce the intended application, particularly for tribocorrosion, biomedical, and photocatalytic systems.

### 6.5. Scale-Up, Bath Management, and Sustainability

Scale-up is likely to be controlled as much by reactor hydrodynamics as by particle chemistry. Larger components introduce longer flow paths, orientation effects, dead zones, non-uniform temperature distributions, and non-uniform electric fields, all of which can change particle availability near the surface. Monitoring particle size or turbidity together with pH, conductivity, and temperature would help determine whether bath composition remains reproducible during repeated processing.

### 6.6. Predictive and Data-Driven Design

Data-driven optimization may become useful once datasets include physically meaningful descriptors such as effective in-bath particle size, zeta potential, particle density, waveform, discharge statistics, and mixing conditions. Models should report predictive uncertainty and be validated across different substrates and reactor scales. Datasets that include null or adverse outcomes would be particularly valuable because the published literature is biased toward positive results.

**Figure 9:**
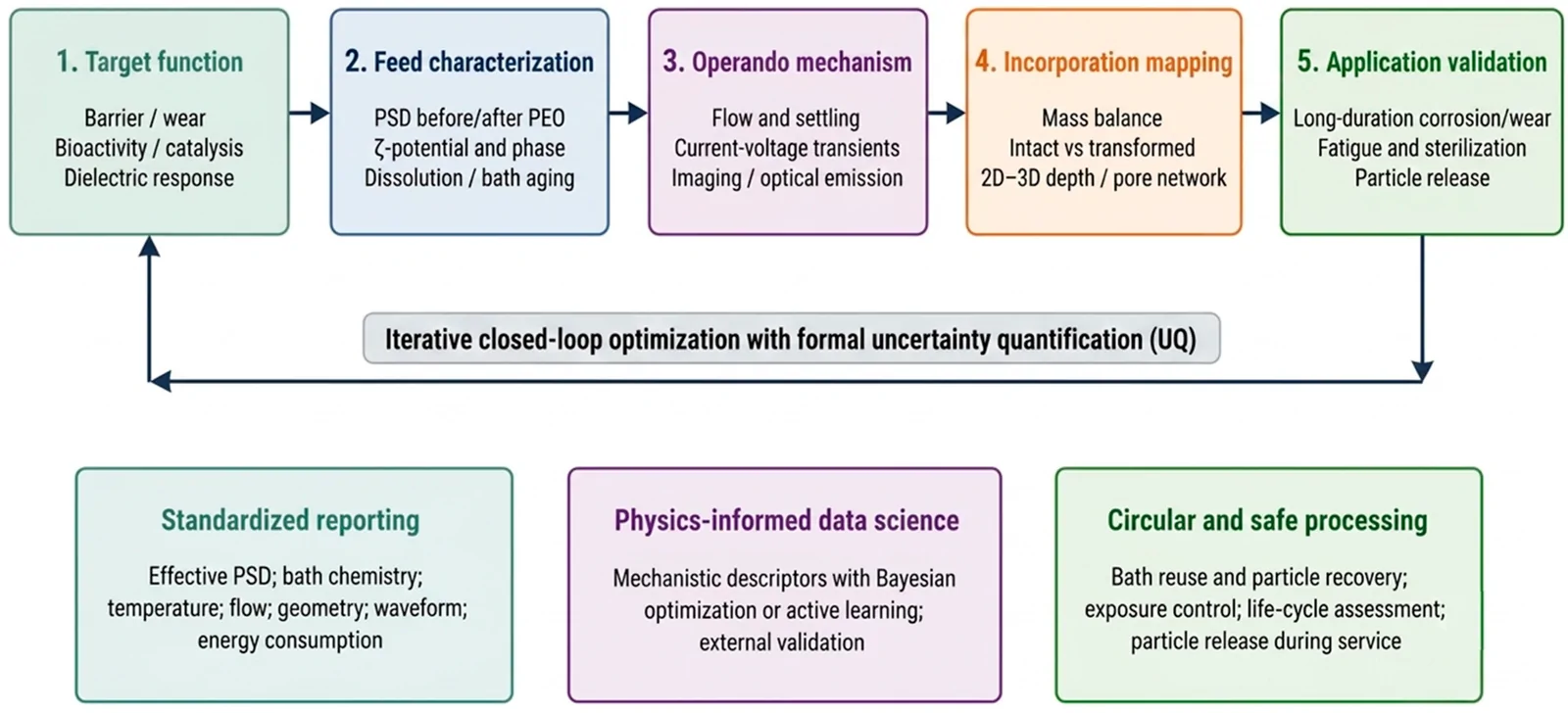
Research and scale-up roadmap for size-controlled particle-assisted PEO, integrating feed characterization, operando diagnostics, quantitative incorporation mapping, application-specific validation, standardized reporting, data-driven optimization, and safe circular processing.

Figure 9 summarizes the main research priorities identified in this review, from characterization of the actual particle feed and operando analysis of transport and discharge behavior to quantitative incorporation measurements and application-specific durability testing. Addressing these measurement and validation gaps would make size-dependent comparisons more reproducible across laboratories and reactor scales.

## 7. Conclusions

Particle size influences every stage of particle-assisted PEO, from suspension stability and delivery to discharge perturbation, transient melt interaction, pore capture, phase formation, and final depth distribution. Well-dispersed submicrometer particles generally remain more uniformly available in the electrolyte, access more limited active pores, equilibrate thermally more rapidly, and react or dissolve more readily. Microparticle transport is governed more strongly by convection and sedimentation and is commonly retained as partially fused inclusions in large craters or the outer porous layer.

The available evidence does not support a general preference for nanoscale additives. Agglomeration can shift the effective size of a nanopowder into the micrometer range, while chemistry, surface charge, density, morphology, concentration, electrolyte composition, substrate, and waveform may outweigh the effect of nominal diameter. Smaller particles are advantageous when uniform pore access and reactive incorporation are required, whereas larger particles may be preferable when retention of the original functional phase or filling of large defects is desired. In either case, excessive loading or poor particle–matrix compatibility can introduce new defects and offset the intended benefit.

The strongest evidence for size-dependent incorporation comes from direct nano–micro comparisons and mechanistic studies showing greater mass uptake and deeper distribution as effective size decreases. Nevertheless, elemental enrichment must not be equated with intact-particle incorporation. A rigorous interpretation requires phase-sensitive, chemical-state, and three-dimensional characterization together with discharge and suspension measurements.

Future studies should prioritize characterization of the effective in-bath particle size, synchronized analysis of discharge behavior, quantitative particle mass balance and depth profiling, and long-term application-specific testing. These measurements would provide a stronger basis for selecting nano-, submicrometer-, micro-, or bimodal particle populations according to application requirements for corrosion protection, tribology, dielectric response, catalysis, optical functionality, and biomedical use.

## Figures and Tables

**Table 1:** Representative electrochemical and interfacial reactions relevant to PEO coating formation.

Electrochemical Reaction	Process Description	Region/Condition
Me → Me^n+^ + ne^−^	Anodic oxidation/dissolution of substrate metal	Anode
xMe + 2yOH^−^ → Me_x_Oᵧ + yH_2_O + 2ye^−^	Generalized anodic oxide formation	Anode; alkaline electrolyte
Me^n+^ + nOH^−^ → Me(OH)_n_	Possible metal-hydroxide precipitation	Near-anode/electrolyte; alkaline conditions
4OH^−^ → O_2_ + 2H_2_O + 4e^−^	Anodic oxygen evolution	Anode; alkaline electrolyte
2H_2_O + 2e^−^ → H_2_ + 2OH^−^	Cathodic hydrogen evolution	Cathode; alkaline/neutral electrolyte

**Table 2:** Size-dependent tendencies, key preconditions, and principal risks associated with nanoparticle and microparticle additions in PEO.

Aspect	Size-Dependent Tendency	Key Precondition	Main Risks/Qualifications	Refs.
Suspension and delivery	Nano: longer colloidal residence, higher number density, and greater contribution from Brownian motion. Micro: transport increasingly governed by convection, sedimentation, and component orientation.	Stable dispersion, suitable zeta potential, adequate mixing, and control of effective agglomerate size.	Nanoparticle agglomeration can shift the effective size into the micrometer range, while microparticle settling can create strong spatial variations in particle availability.	[69,146]
Discharge interaction	Nano: potentially more spatially distributed modification of discharge activity. Micro: more localized electrical and thermal heterogeneities and possible concentration of discharge activity around individual inclusions.	Appropriate particle concentration and consideration of particle conductivity, dielectric properties, waveform, and electrical control mode.	Excessive loading or conductive particle networks can destabilize discharges; the direction of the effect is not determined by size alone.	[77,137,138]
Melt and phase response	Nano: faster thermal equilibration and a greater likelihood of dissolution, interfacial reaction, and nucleation. Micro: greater probability of partial fusion, surface reaction, or retention of an intact core.	Sufficient particle–melt contact and favorable chemistry, wetting, thermal properties, and discharge energy.	High thermal stability may suppress reaction even for small particles, whereas sufficiently energetic discharges can transform coarse particles; size alone therefore does not determine phase retention.	[88,112,145]
Depth distribution	Nano: greater probability of entering fine pores and producing deeper or more continuous particle-derived profiles. Micro: preferential retention in large pores, discharge craters, and outer coating regions.	Stable suspension and particle/agglomerate dimensions smaller than accessible pore or channel dimensions.	Elemental enrichment at depth does not demonstrate penetration of intact particles because dissolution, reaction, and redistribution of particle-derived species may produce similar profiles.	[64,142,146,153]
Corrosion resistance	Nano: fine-pore sealing and formation of compact or protective reaction products may improve barrier behavior. Micro: filling of large defects and introduction of protective phases may also improve corrosion resistance.	Adequate particle dispersion, incorporation, interfacial bonding, and a net reduction in connected defects.	Agglomerates, interfacial gaps, cracks, connected porosity, or galvanically active phases can negate or reverse the expected improvement.	[141,144,154]
Wear and friction	Nano: more uniform hardening, pore filling, or distribution of solid-lubricating phases. Micro: localized reinforcement or retention of relatively intact functional particles.	Strong particle–matrix bonding and suitable hardness, lubrication behavior, surface roughness, and particle retention.	Poorly bonded particles can detach, promote cracking, or act as abrasive third-body debris; excessive hard-particle addition may also increase roughness.	[145,149,154,155]
Biofunctional response	Nano: high specific surface area and more homogeneous functional distribution can enhance interfacial reactions, ion release, antibacterial activity, or catalytic/photocatalytic response. Micro: greater phase retention and potential for more localized or slower functional release.	Controlled loading, stable incorporation, appropriate particle chemistry, and preservation of the intended functional phase.	Excessive release, cytotoxicity, particle detachment, phase transformation, or loss of functionality during service can outweigh the intended benefit.	[121,153,159]

## Data Availability

No new data were created or analyzed in this study. Data sharing is not applicable to this article.
